# Identification of a 5-Gene-Based Scoring System by WGCNA and LASSO to Predict Prognosis for Rectal Cancer Patients

**DOI:** 10.1155/2021/6697407

**Published:** 2021-03-23

**Authors:** He Huang, Shilei Xu, Aidong Chen, Fen Li, Jiezhong Wu, Xusheng Tu, Kunpeng Hu

**Affiliations:** ^1^General Surgery Department, The Third Affiliated Hospital of Sun Yat-sen University, Guangzhou 510530, China; ^2^Department of Physiology, Nanjing Medical University, Nanjing 211166, China; ^3^Department of Emergency Medicine, The Third Affiliated Hospital of Sun Yat-sen University, Guangzhou 510530, China

## Abstract

**Background:**

Although accumulating evidence suggested that a molecular signature panel may be more effective for the prognosis prediction than routine clinical characteristics, current studies mainly focused on colorectal or colon cancers. No reports specifically focused on the signature panel for rectal cancers (RC). Our present study was aimed at developing a novel prognostic signature panel for RC.

**Methods:**

Sequencing (or microarray) data and clinicopathological details of patients with RC were retrieved from The Cancer Genome Atlas (TCGA-READ) or the Gene Expression Omnibus (GSE123390, GSE56699) database. A weighted gene coexpression network was used to identify RC-related modules. The least absolute shrinkage and selection operator analysis was performed to screen the prognostic signature panel. The prognostic performance of the risk score was evaluated by survival curve analyses. Functions of prognostic genes were predicted based on the interaction proteins and the correlation with tumor-infiltrating immune cells. The Human Protein Atlas (HPA) tool was utilized to validate the protein expression levels.

**Results:**

A total of 247 differentially expressed genes (DEGs) were commonly identified using TCGA and GSE123390 datasets. Brown and yellow modules (including 77 DEGs) were identified to be preserved for RC. Five DEGs (ASB2, GPR15, PRPH, RNASE7, and TCL1A) in these two modules constituted the optimal prognosis signature panel. Kaplan-Meier curve analysis showed that patients in the high-risk group had a poorer prognosis than those in the low-risk group. Receiver operating characteristic (ROC) curve analysis demonstrated that this risk score had high predictive accuracy for unfavorable prognosis, with the area under the ROC curve of 0.915 and 0.827 for TCGA and GSE56699 datasets, respectively. This five-mRNA classifier was an independent prognostic factor. Its predictive accuracy was also higher than all clinical factor models. A prognostic nomogram was developed by integrating the risk score and clinical factors, which showed the highest prognostic power. ASB2, PRPH, and GPR15/TCL1A were predicted to function by interacting with CASQ2/PDK4/EPHA67, PTN, and CXCL12, respectively. TCL1A and GPR15 influenced the infiltration levels of B cells and dendritic cells, while the expression of PRPH was positively associated with the abundance of macrophages. HPA analysis supported the downregulation of PRPH, RNASE7, CASQ2, EPHA6, and PDK4 in RC compared with normal controls.

**Conclusion:**

Our immune-related signature panel may be a promising prognostic indicator for RC.

## 1. Introduction

Previously, colon cancer (CC) and rectal cancer (RC) are considered a single tumor entity (called colorectal cancer (CRC)) [1]. However, recent studies indicate that there are significant differences in epidemiology, pathology, molecular mechanisms, and the response to treatments [1]. The risk of developing RC is estimated to be four times higher than that of CC, and RC patients have a lower 5-year survival rate than CC patients (60% versus 72%) due to a poor response to current treatment options [1–3]. Thus, it is of particular importance to explore approaches to early separate RC patients with a high death risk and then provide improved specialized care to further reduce the overall mortality rate.

With the developments in sequencing technology and bioinformatics, recent scholars suggest that a molecular signature panel may be more effective for the prognosis prediction than routine clinical characteristics (such as the tumor-node-metastasis (TNM) stage) [4, 5]. Li et al. identified a four-mRNA signature panel as an independent prognostic factor for CRC. This four-mRNA signature panel can effectively predict the prognosis of CRC patients, with an area under the receiver operating characteristic (ROC) curve (AUC) of 0.730. The stratified analysis indicated that the patients belonging to the same T stage (T3+T4), N stage (N1+N2), or TNM stage (III+IV) can also be stratified into the high-risk and low-risk groups using this 4-gene signature panel [6]. The study of Zuo et al. revealed that a six-mRNA signature panel had a significant prognostic value to discriminate the high-risk patients from the low-risk patients, with an AUC of 0.711 and 0.683 for 3-year and 5-year survival, respectively. This 6-gene signature panel was an independent factor of OS after adjustment for clinical factors and can predict different survival outcomes in patients with the early (or advanced) TNM stage [7]. Sun et al. developed a 12-gene expression signature panel to precisely predict the prognosis for CC patients, which could distinguish poor from good prognosis patients within stage II/III [8]. Chen et al. found that the signature panel consisting of 16 gene pairs formed by 24 genes had a better prognostic ability than the TNM stage (AUC: 0.724 vs. 0.703; concordance index (C-index): 0.869 vs. less than 0.8) at 5 years [9]. Although there were also studies to identify mRNAs associated with the prognosis of RC patients [10–13], no reports specifically focused on the signature panel and compared its prognostic values with clinical factors.

In the present study, we aimed to (1) screen RC-related genes by weighted gene coexpression network analysis (WGCNA) [14], (2) develop a reliable mRNA signature panel for the prediction of overall survival (OS) in patients with RC using the least absolute shrinkage and selection operator (LASSO) method [15, 16], (3) validate its superior prognostic performance to various clinical features by stratified analysis and comparison of the AUC and C-index, and (4) establish a clinicopathologic-mRNA nomogram to improve the prediction accuracy clinically.

## 2. Materials and Methods

### 2.1. Data Access

**Figure 1:**
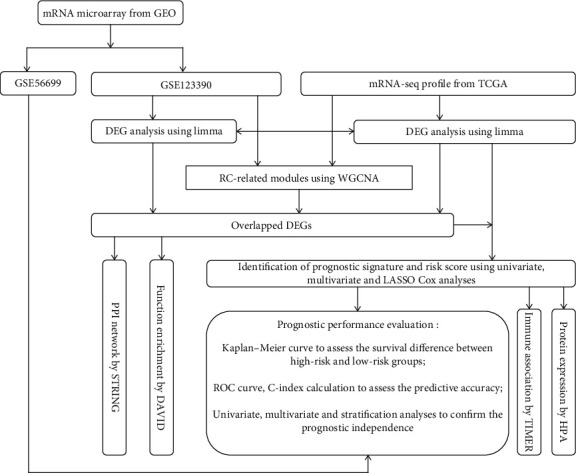
A study flowchart for our analysis.

The RNA-seq expression data (fragments mapped per kilobase of exon per million reads mapped, level 3) were retrieved from The Cancer Genome Atlas (TCGA; https://portal.gdc.cancer.gov) database using “TCGA-rectal adenocarcinoma (READ)” as the keyword. There were 177 READ cases and 10 controls in this dataset. However, only 162 READ cases provided clinical information. Thus, TCGA dataset (including 162 cases and 10 controls) served as the training dataset for our following analyses. Furthermore, GSE123390 (platform: Affymetrix Human Transcriptome Array 2.0) and GSE56699 (platform: Illumina HumanHT-12 WG-DASL V4.0 R2 expression beadchip) microarray datasets were also obtained from the Gene Expression Omnibus (GEO, http://www.ncbi.nlm.nih.gov/geo/) database using “rectal cancer” as the keyword. GSE123390 was used for the WGCNA model validation because it investigated the mRNA expression profile in human RC tissues (*N* = 28) and controls (*N* = 5). GSE56699 was used for the survival model validation because it provided the prognosis information for 61 of 72 RC cases. The study flowchart is illustrated in Figure 1.

### 2.2. Identification of Differentially Expressed Genes (DEGs)

In TCGA-READ and GSE123390 datasets, the DEGs were screened between RC and controls using the limma package of R (version 3.34.7; https://bioconductor.org/packages/release/bioc/html/limma.html) [17]. False discovery rate (FDR) < 0.05 and ∣log_2_FC (fold change) | >0.5 were defined as the statistical threshold. All DEGs in these two datasets were subjected to the hierarchical clustering analysis using the pheatmap package of R (version 1.0.8; https://cran.r-project.org/web/packages/pheatmap), and the heat map generated was used to assess the heterogeneity of gene expression patterns between RC and controls. The Draw Venn Diagram online tool (http://bioinformatics.psb.ugent.be/webtools/Venn) was utilized to identify the shared DEGs between TCGA-READ and GSE123390 datasets. The functions of common DEGs were analyzed using the gProfiler tool (http://biit.cs.ut.ee/gprofiler/gost). Gene Ontology (GO) terms, Kyoto Encyclopedia of Genes and Genomes (KEGG), and Reactome pathways with an FDR < 0.05 were considered to be statistically significant.

### 2.3. WGCNA

To screen genes associated with the development of RC, WGCNA was performed using the WGCNA package in R (version 1.61; https://cran.r-project.org/web/packages/WGCNA/index.html) [14], by which highly correlated mRNAs could be clustered into the same coexpression modules. WGCNA included six steps: (1) calculation of the expression and connectivity correlations of mRNAs between TCGA-READ and GSE123390 datasets; (2) selection of the soft threshold power (*β*) according to the scale-free topology criterion; (3) calculation of the topological overlap matrix dissimilarity between genes in TCGA-READ to build the dendrogram and identification of modules (cutHeight = 0.995 and minSize ≥ 50) by the Dynamic Tree Cut method [18]; (4) assessment of the preservation (*Z*‐score > 5 and *p* < 0.05) of modules in two datasets using the modulePreservation statistics [19]; (5) enrichment of DEGs to modules using the hypergeometric algorithm [*f*(*k*, *N*, *M*, *n*) = *C*(*k*, *M*)∗*C*(*n* − *k*, *N* − *M*)/*C*(*n*, *N*)] [20]; and (6) association of coexpression modules with clinical information.

### 2.4. Protein-Protein Interaction (PPI) Network

The PPI pairs among DEGs in crucial modules were identified using the Search Tool for the Retrieval of Interacting Genes (STRING; version 11.0; https://string-db.org) database [21]. Only interactions with a combined score > 0.4 were selected to construct the PPI network using Cytoscape software (version 3.4; http://www.cytoscape.org/) [22]. The functions of genes in the PPI network were analyzed using the KEGG, GO, and Reactome implements in the STRING. Significant GO terms, KEGG pathways, and Reactome pathways were chosen using an FDR < 0.05 as the statistical threshold.

### 2.5. Development of the Prognostic Signature Panel

The univariate Cox regression analysis was used to screen OS-associated genes from the DEGs in the preserved modules. The DEGs with a log-rank *p* < 0.05 in the univariate analysis were entered into the multivariate Cox regression model to identify independent predictors. An L1-penalized (LASSO) Cox proportional hazard model in the penalized package (version 0.9-5; http://bioconductor.org/packages/penalized/) [15, 16] was further applied on these independent prognostic DEGs to select the optimal subset of signature panels. The risk score model was established based on the expression levels of prognostic DEGs (Exp_DEGs_) and their LASSO coefficients (*β*_DEGs_):

(1) $$\begin{array}{c} \text{Risk score}=\beta_{\text{mRNA}1}\times {\text{Exp}}_{\text{mRNA}1}+\cdots \beta_{\text{mRNA}n}\times {\text{Exp}}_{\text{mRNA}n}. \end{array}$$

The patients were divided into the high-risk group and the low-risk group by using the median risk score as the cutoff. The OS differences between the high-risk group and the low-risk group were compared according to the Kaplan-Meier survival curve analysis and log-rank test. The predictive accuracy of the risk score was estimated through the AUC calculated from the ROC curve. These analyses were first carried out for TCGA-READ dataset and then validated in the GSE56699 dataset.

Moreover, univariate and multivariate Cox analyses were applied using TCGA-READ cohort to evaluate whether the risk score was independent of other clinical variables for the prognosis prediction. Kaplan-Meier survival curve analysis was used to identify whether the risk score was also an effective tool for stratification of patients with the same clinical characteristics. A nomogram that incorporated the risk score and clinical prognostic factors was developed for the prediction of 3-year and 5-year OS rates. The predictive power of the nomogram was assessed in terms of the AUC and the C-index (which was calculated using the survcomp package, http://www.bioconductor.org/packages/release/bioc/html/survcomp.html).

### 2.6. Analysis of Immune Cell Infiltration

The associations between the expression of selected prognostic genes and the abundance of six tumor-infiltrating immune cells in TCGA-PRAD (B cells, CD4+ T cells, CD8+ T cells, neutrophils, macrophages, and dendritic cells) were estimated from the Tumor Immune Estimation Resource (TIMER; https://cistrome.shinyapps.io/timer/) based on Spearman's correlation test. *p* < 0.05 adjusted by the tumor purity was considered significant.

### 2.7. Verification of Protein Expressions of Prognostic Genes

The immunohistochemical results for the prognostic-related DEGs and their interaction genes in rectal and normal tissues were downloaded from the Human Protein Atlas (HPA) to confirm their protein expression levels.

## 3. Results

### 3.1. DEGs between RC and Normal Controls

**Figure 2:**
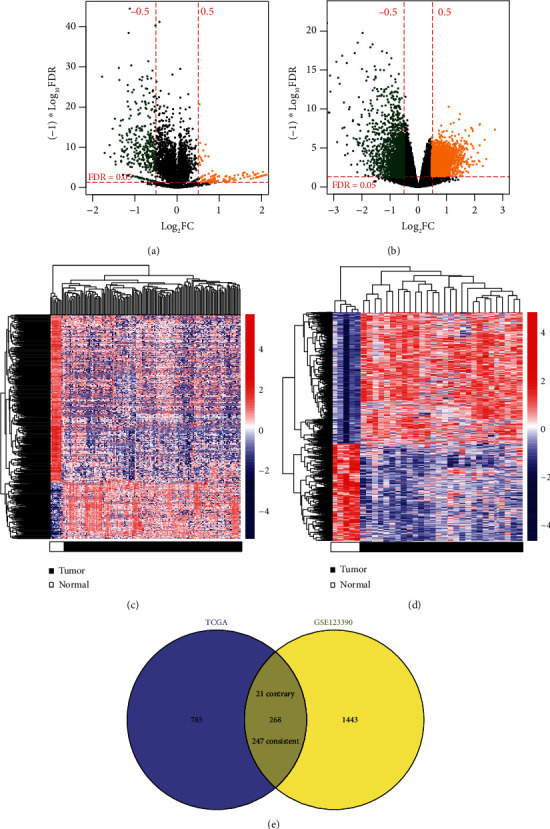
Identification of differentially expressed mRNAs in two datasets. (a) A volcano plot of differentially expressed RNAs between 162 rectal cancer tissues and 10 normal control tissues of TCGA dataset. (b) A volcano plot of differentially expressed RNAs between 28 rectal cancer tissues and 5 normal control tissues of the GSE123390 dataset. (c) A heat map of differentially expressed RNAs in TCGA dataset. (d) A heat map of differentially expressed RNAs in the GSE123390 dataset. (e) A Venn diagram to display the overlapped differentially expressed RNAs between two datasets. Yellow, high expression; green, low expression. FC: fold change; FDR: false discovery rates; TCGA: The Cancer Genome Atlas.

Based on the threshold of ∣log_2_FC | >0.5 and FDR < 0.05, 1053 DEGs (including 830 downregulated and 223 upregulated) were screened between 162 RC tissues and 10 normal control tissues of TCGA dataset, while 1711 DEGs (including 728 downregulated and 983 upregulated) were identified between 28 RC tissues and 5 normal control tissues of the GSE123390 dataset. The volcano plot and heat map of these two datasets are shown in Figures 2(a) and 2(b) and Figures 2(c) and 2(d), respectively. In addition, the Draw Venn Diagram online tool was used to investigate the intersection of DEGs in these two datasets. As a result, 268 overlapped DEGs were found, but the expression trend was consistent only in 247 DEGs (Figure 2(e); Table S1).

These 247 genes were subjected to the gProfiler online toolset for the function enrichment analysis. The results showed that 9 GO molecular function terms (such as glycosaminoglycan binding: ribonuclease A family member 7 (RNASE7)), 57 GO biological process terms (such as response to endogenous stimuli: calsequestrin 2 (CASQ2), pyruvate dehydrogenase kinase 4 (PDK4), and C-X-C motif chemokine ligand 12 (CXCL12); ion transport: CASQ2 and CXCL12; response to organic substance: CASQ2, TCL1 family AKT coactivator A (TCL1A), and CXCL12; response to chemical: PDK4 and EPH receptor A6 (EPHA6); chemotaxis: CXCL12 and EPHA6; and regulation of heart contraction: CASQ2), 7 KEGG pathways (such as calcium signaling pathway: CASQ2), and 5 Reactome pathways (such as muscle contraction: CASQ2; GPCR ligand binding: CXCL12) were enriched (Table S2).

### 3.2. Identification of RC-Related Modules by WGCNA

**Figure 3:**
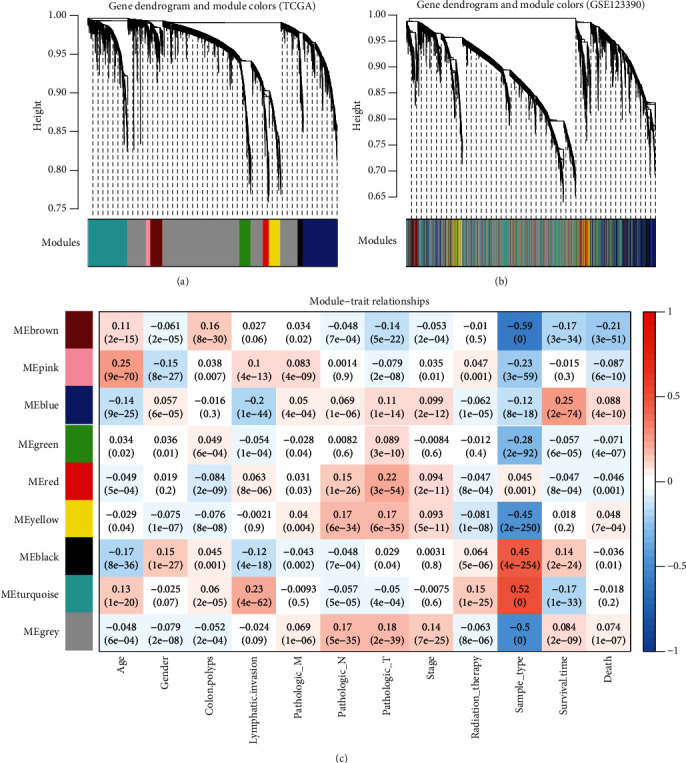
Coexpression modules screened based on the WGCNA. (a) A clustering dendrogram of coexpression modules screened using TCGA dataset. (b) A dendrogram of coexpression modules screened using the GSE123390 dataset. (c) The association between modules and clinical information of rectal patients.

The correlation analysis showed that there were positive correlations in the expression level (cor = 0.46, *p* < 1*e* − 200) and the connectivity (cor = 0.23, *p* < 8.1*e* − 100) of RNAs between the training dataset TCGA and the validation dataset GSE123390. Using TCGA dataset, the soft threshold power 10 was chosen to create networks with a scale-free topology (*R*^2^ reached 0.9 for the first time; the mean connectivity was equal to 1). The dendrogram displayed that the genes were clustered into 9 modules using TCGA dataset (Figure 3(a)). These modules were also validated in the analysis of the GSE123390 dataset (Figure 3(b)). Among them, blue, brown, grey, red, turquoise, and yellow modules were considered to be preserved because of their *Z*‐score > 5 and *p* value < 0.05 (Table 1). Brown and yellow modules were significantly enriched by DEGs (enrichment fold > 1 and *p* value < 0.05), suggesting they may be particularly crucial for the development of RC (Table 1). The genes in the brown and yellow modules were also found to be significantly associated with the pathologic M, pathologic N, pathologic T, pathologic stage, survival time, and death of RC patients (Figure 3(c)).

### 3.3. Construction of a PPI Network

The 77 DEGs in the brown and yellow modules were uploaded to the STRING database to obtain their interaction relationships. As a result, 281 interaction pairs between 69 DEGs were identified (such as peripherin- (PRPH-) PTN, ankyrin repeat and SOCS box containing 2- (ASB2-) CASQ2/PDK4/EPHA6, and G protein-coupled receptor 15 (GPR15)/TCL1A-CXCL12), which were used to construct the PPI network (Figure S1). Function enrichment analysis obtained 30 GO biological process terms (such as regulation of heart contraction: CASQ2; regulation of ion transport: CASQ2 and CXCL12; regulation of tissue remodeling: PDK4; negative regulation of leukocyte tethering or rolling: CXCL12; and negative regulation of dendritic cell apoptotic process: CXCL12), 4 KEGG pathways (such as metabolism of xenobiotics by cytochrome P450 and chemical carcinogenesis), and 4 Reactome pathways (such as muscle contraction: CASQ2) (Table S3).

### 3.4. Development of a Prognostic Risk Score

The univariate Cox regression analysis identified that 35 DEGs were significantly associated with OS, including EPHA6 (hazard ratio (HR) = 1.11), CASQ2 (HR = 1.12), and PDK4 (HR = 1.45) (Table S4). Eight of them were screened as independent prognostic predictors after the multivariate Cox regression analysis (Table S5). The use of a LASSO-based Cox PH model further identified that 5 DEGs (ASB2, GPR15, PRPH, RNASE7, and TCL1A) might constitute the optimal signature panel for the prognosis prediction (Table 2).

**Figure 4:**
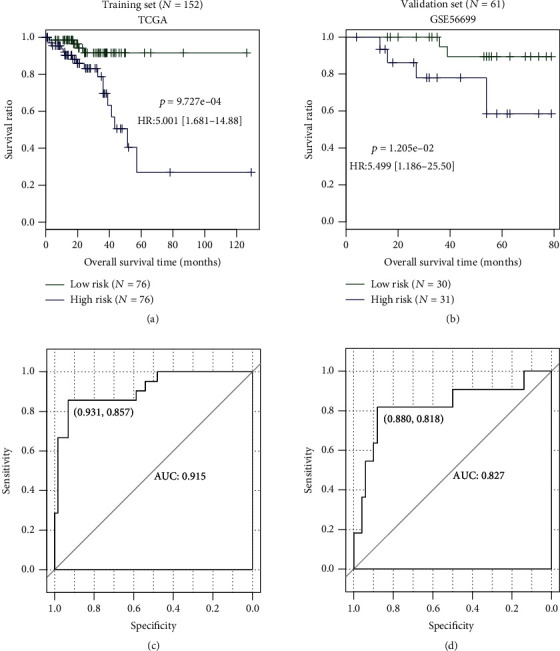
The prognostic performance of the 5-gene risk score model. (a, b) The Kaplan-Meier survival curve to show the overall survival differences of patients with the high-risk score and the low-risk score in TCGA (a) and GSE56699 (b) datasets. (c, d) The ROC to demonstrate the prognostic accuracy of the risk score for the overall survival of patients in TCGA (c) and GSE56699 (d) datasets. TCGA: The Cancer Genome Atlas; HR: hazard ratio; AUC: area under the receiver operating characteristic (ROC) curve.

The prognostic risk score was estimated for each patient in the training TCGA dataset and the validation GSE56699 dataset by the following formula: (0.3082 × expression of ASB2) + (−0.0651 × expression of GPR15) + (0.0701 × expression of PRPH) + (−0.1112 × expression of RNASE7) + (−0.1428 × expression of TCL1A). Based on the median value of the risk score, the patients were divided into the low-risk group and the high-risk group. Kaplan-Meier curve analysis showed that patients in the high-risk group had a significantly poorer prognostic outcome than those in the low-risk group (TCGA: HR = 5.001, 95% confidence interval (CI) = 1.681-14.88, and *p* = 9.727*e* − 04, Figure 4(a); GSE56699: HR = 5.499, 95% CI = 1.186-25.50, and *p* = 1.205*e* − 02, Figure 4(b)). ROC curve analysis demonstrated that this risk score had high predictive accuracy for unfavorable prognosis, with the AUC of 0.915 (Figure 4(c)) and 0.827 (Figure 4(d)) for TCGA and GSE56699 datasets, respectively.

**Figure 5:**
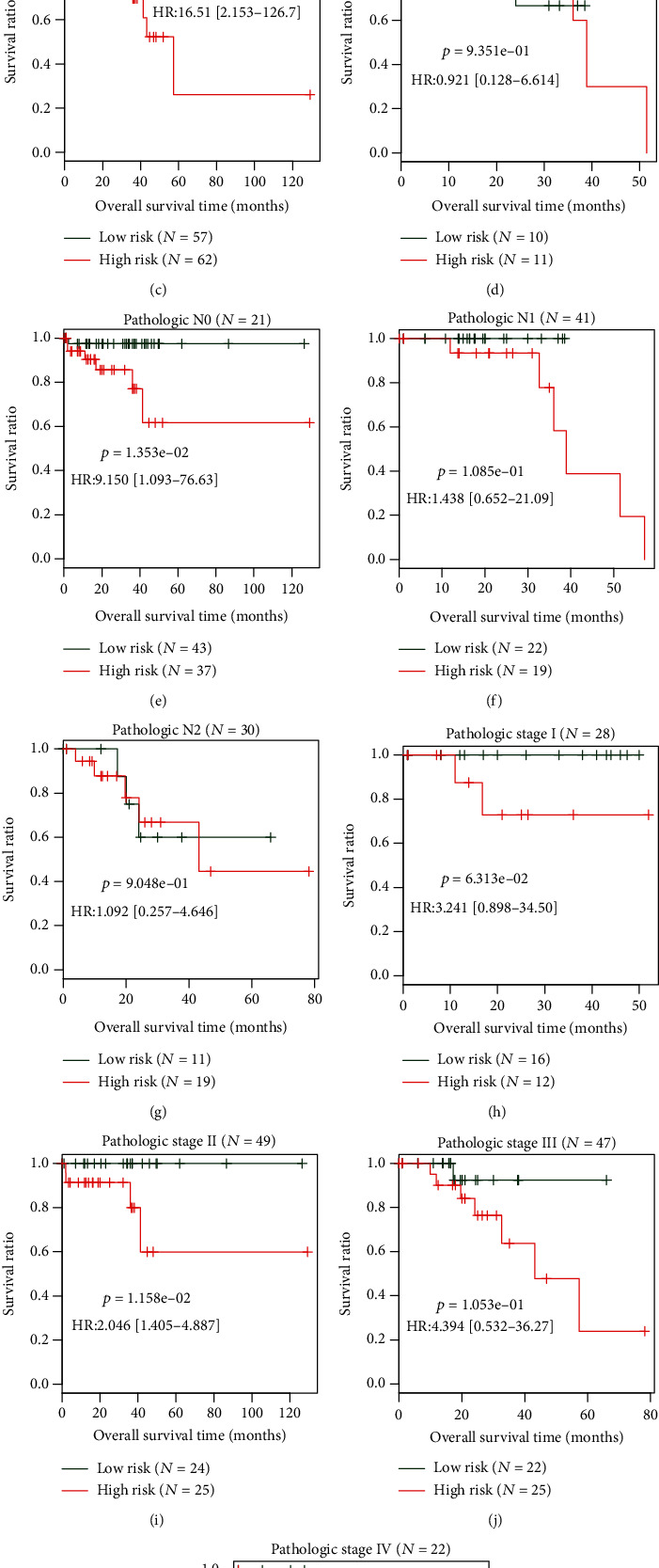
Risk stratification models based on age, pathologic M, pathologic N, and pathologic stage. (a, b) Age stratification (>65 years and <65 years). (c, d) Pathologic M stratification (M0 and M1). (e–g) Pathologic N stratification (N0, N1, and N2). (h–k) Pathologic stage stratification (I, II, III, and IV). HR: hazard ratio.

**Figure 6:**
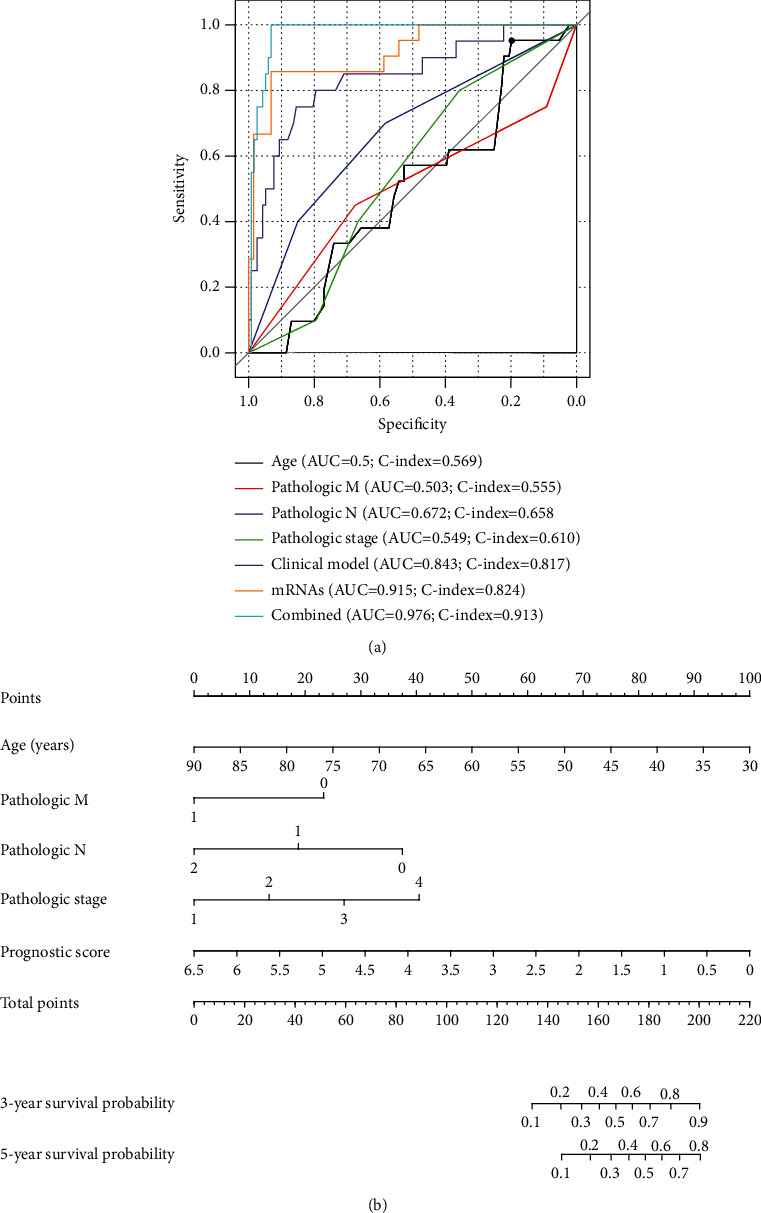
A prognostic nomogram to predict the survival probability of patients with rectal cancer. (a) Receiver operating characteristic curve to demonstrate the superiority of the risk score for the prognosis prediction to other clinical factors. (b) A prognostic nomogram. AUC: area under the receiver operating characteristic curve; C-index: concordance index.

Univariate and multivariate Cox regression analyses were also performed using the risk score and other clinical factors to confirm the independence of our five-mRNA signature panel. The results displayed that after multivariable adjustments for clinicopathological factors, the risk score remained significantly associated with patients' OS (Table 3), suggesting this five-mRNA-based classifier was an independent prognostic factor. In addition, the risk score was shown to have the ability to further classify the population with age above 65 years (*p* = 2.606*e* − 03), pathologic M0 (*p* = 1.013*e* − 04), pathologic N0 (*p* = 1.353 − 02), and pathologic stage II (*p* = 1.158*e* − 02) and predict their different prognosis results (Figure 5), indicating its prognostic superiority to routine clinical factors. Moreover, the time-dependent ROC curve analysis also demonstrated that the predictive accuracy of the risk score (AUC = 0.915; C-index = 0.824) was higher than that of age (AUC = 0.5; C-index = 0.569), pathologic M (AUC = 0.503; C-index = 0.555), pathologic N (AUC = 0.672; C-index = 0.658), and pathologic stage (AUC = 0.549; C-index = 0.610) and the model with all clinical factors (AUC = 0.843; C-index = 0.817) (Figure 6(a)). Therefore, the risk score should be integrated with the clinical factors to better predict the prognosis clinically, based on which a prognostic nomogram was developed (Figure 6(b)). As expected, the AUC (0.976) and the C-index (0.913) of the nomogram were higher than those of any clinical factor model and the risk score model (Figure 6(a)).

### 3.5. Correlations between mRNA Levels of Prognostic Genes and Tumor-Infiltrating Immune Cells

**Figure 7:**
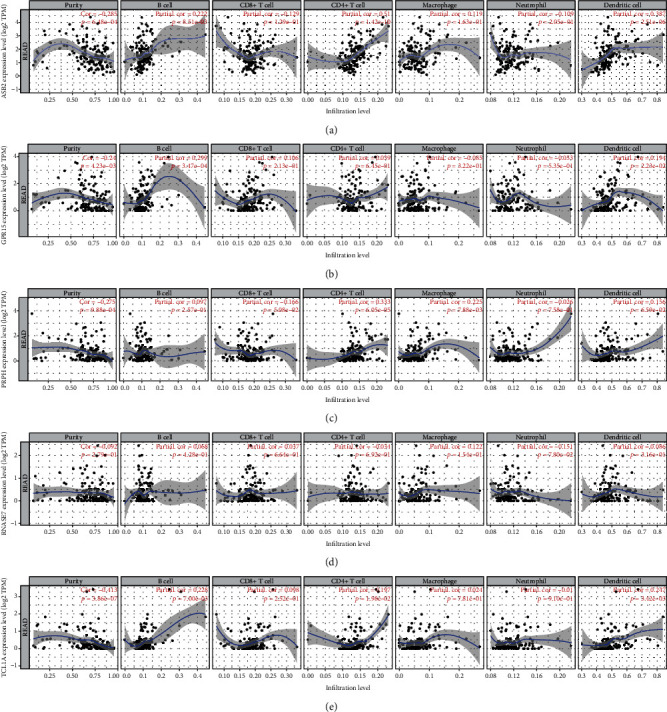
Correlation of mRNA levels of prognostic genes and infiltration levels of immune cells in TCGA-READ samples using the TIMER database. (a) ASB2. (b) GPR15. (c) PRPH. (d) RNASE7. (e) TCL1A. TCGA: The Cancer Genome Atlas; READ: rectal adenocarcinoma; TIMER: Tumor Immune Estimation Resource; ASB2: ankyrin repeat and SOCS box containing 2; GPR15: G protein-coupled receptor 15; PRPH: peripherin; RNASE7: ribonuclease A family member 7; TCL1A: TCL1 family AKT coactivator A.

TIMER analysis revealed that there was a significant correlation between ASB2/TCL1A expression and B cells, CD4+ T cells, and dendritic cells; the expression of GPR15 was positively associated with the abundance of B cells and dendritic cells (DCs); the expression of PRPH positively correlated with the infiltration levels of CD4+ T cells and macrophages (Figure 7). No significant association was observed between the expression of RNASE7 and infiltration levels of all six immune cells (Figure 7).

### 3.6. Validation of Protein Expressions of Prognostic Genes

**Figure 8:**
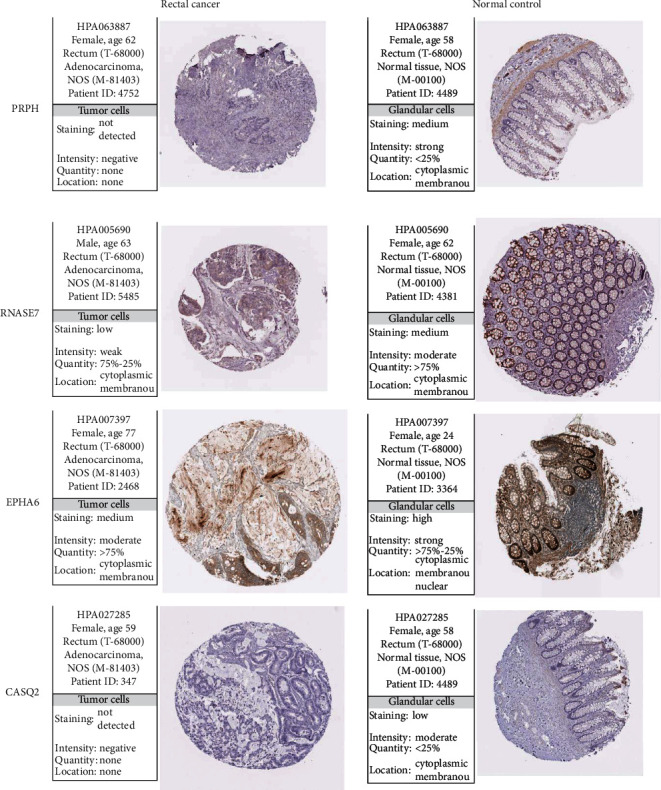
Protein expression of prognostic genes and their interaction genes validated using the online HPA database.

The expressions of 5 signature genes and their interaction genes were validated using the immunostaining results from the HPA database. The results supported the downregulation of PRPH, RNASE7, CASQ2, EPHA6, and PDK4 in RC compared with normal controls (Figure 8). GPR15, TCL1A, and PTN were not detected in both RC tissues and normal rectal tissues. There was no evidence of immunostaining for ASB2 and CXCL12. No rectal samples were collected to investigate the expression of PDK4 in RC.

## 4. Discussion

In the present study, we established a risk score model based on five prognostic DEGs (ASB2, GPR15, PRPH, RNASE7, and TCL1A). ROC curve analysis indicated that this 5-mRNA signature panel can accurately predict the prognosis for patients with RC, with the AUC of 0.915 and 0.827 for the training and validation datasets, respectively. The prognostic performance of our risk score seemed to be better than that of previously reported signature panels developed for CRC (such as 4 genes: AUC = 0.722 for the external dataset and 0.607 for the internal dataset [4]; 6 genes: AUC = 0.683 [7]; and 9 genes: AUC = 0.741 [23]) or CC (16 gene pairs: AUC = 0.724 [9]; 9 genes: AUC = 0.676 [22]). In addition, in the study of Zuo et al. [7], they performed a subgroup analysis to confirm whether the 6-gene signature panel was effective for colon adenocarcinoma (COAD) and READ. As a result, the AUC was, respectively, 0.653 and 0.74 for COAD and READ, which were both lower than that of our signature panel. Moreover, in line with other signature panels identified for CRC or CC patients [6–9, 24], our risk score was demonstrated to be an independent factor for the prognosis prediction and stratify the survival of patients with the same TNM stage. Also, the AUC and the C-index of the risk score were higher than those of age (AUC = 0.5; C-index = 0.569), pathologic M (AUC = 0.503; C-index = 0.555), pathologic N (AUC = 0.672; C-index = 0.658), and pathologic stage (AUC = 0.549; C-index = 0.610) and the model with all clinical factors (AUC = 0.843; C-index = 0.817). These findings reveal that our risk score may serve as an effective molecular biomarker to predict the poor prognosis of patients with RC. To better guide prognostication in clinical practice, some authors suggest that molecular prognostic models and clinicopathological models should be combined [24–27], which showed the highest predictive power compared with anyone. In agreement with these studies, our results also showed that the nomogram that integrated the five-mRNA classifier and four clinical risk factors (age, pathologic M, pathologic N, and pathologic stage) had the highest AUC (0.976) and C-index (0.913).

Although all of the 5 signature genes were not included in the previous signature panels for CRC [6–9, 24], some of them were found to be associated with the progression and prognosis of CRC (including ASB2, GPR15, TCL1A, and PRPH) [28–31]. High ASB2 expression was shown to predict a short relapse-free survival for patients with CRC [28]. Deletion of ASB2 in hematopoietic cells inhibited the shortening of the colon and the tumor load in mice. Function analysis indicated that ASB2 may exert tumor-promoting roles by decreasing Th1, Th17, and cytotoxic CD8+ T cell response which are beneficial for protection against tumor progression [28]. Consistent with the study of Spinner et al. [28], our results also demonstrated that patients with a high level of ASB2 may have a 12.505-fold higher risk of possessing a worse OS rate than those with low expression. Also, ASB2 was predicted to interact with the chemotaxis-associated EPHA6 gene which could be hypermethylated to result in downregulated EPHA6 expression by anti-inflammatory interleukin-6 [32, 33], a Th17 cell biomarker [34]. The knockdown of EPHA6 decreased prostate cancer cell invasion *in vitro* and reduced lung and lymph node metastasis *in vivo* [35]. High EPHA6 expression was associated with a lower OS rate in patients with breast cancer [36] and our RC (HR = 1.11). In addition to EPHA6, we also predicted that ASB2 may be involved in RC by influencing the expressions of CASQ2 and PDK4. Highly expressed CASQ2 [37] and PDK4 [38] were reported to be significantly correlated with poor OS and disease-free survival in cancer patients. Overexpression of PDK4 promoted cell proliferation, invasion, and tumor growth *in vivo* [38]. These prognosis conclusions of CASQ2 (HR = 1.12) and PDK4 (HR = 1.45) were also confirmed in our study on RC. TCL1A is crucial for cancer development by expressing in a subpopulation of immune B cells (CD3-/CD19+/CD10+/CD34-). A high TCL1A/CD20 (B cell) ratio or TCL1A expression was shown to correlate with improved survival [39, 40]. In agreement with other cancers [39, 40], our study also showed that TCL1A was a protective risk for the survival of RC patients (HR = 0.530) and positively associated with the abundance of B cells. Furthermore, we predicted TCL1A may interact with CXCL12, a chemokine gene that was speculated to be involved in the regulation of the dendritic cell apoptotic process in our function enrichment analysis. High CXCL12 expression was reported to confer a survival advantage for breast cancer patients [41] and stage III CC [42]. Silencing of CXCL12 by transforming growth factor-*β* in mesenchymal stromal cells of the primary tumor site promoted the tumor metastasis by increasing the expression of CXCR7, a CXCL12 receptor [43]. A meta-analysis showed that immunotherapy with DCs significantly improved OS at 6 months, 1 year, 3 years, and 5 years of patients with hepatocellular carcinoma [44]. Coculture of DCs significantly inhibited liver cancer stem cell growth *in vitro* and *in vivo* [45]. Consistent with these findings, we also found that the expression of TCL1A was significantly positively correlated with the infiltration of DCs. Using TCGA and the genotype-tissue expression data, Wang and Wang found that GPR15 was significantly lowly expressed in COAD and READ compared with normal tissues [30], which was validated in our study. GPR15 expression was significantly positively correlated with the prognosis of patients with COAD (that is, the high expression had a longer OS) [30], which was also observed in our study of READ. Wang and Wang believed that GPR15 may be a tumor suppressor by regulating a serial of genes enriched in immune systems and increasing the infiltration of B cells (in neck squamous carcinoma, lung adenocarcinoma, and stomach adenocarcinoma), CD4+ T cells, and DCs (in neck squamous carcinoma and stomach adenocarcinoma) [30]. Similarly, we found that the expression of GPR15 was positively associated with the levels of tumor-infiltrating immune B cells and DCs and predicted that GPR15 could interact with DC-related CXCL12 to participate in RC progression. CD133+ human umbilical hematopoietic progenitor cells were revealed to promote the proliferation and invasion of CRC cells *in vitro* and enhance tumor growth and metastasis *in vivo* by upregulating PRPH [31]. However, the mechanisms of PRPH in CRC remained unclear. In this study, we speculated that PRPH may function by interacting with downstream PTN. Tumor-associated macrophages increased the proportion of cancer stem cells in lymphoma by secreting PTN [46]. Upregulated PTN promoted tumor cell proliferation and inhibited apoptosis and chemosensitivity by activating the NF-*κ*B pathway [46, 47]. Meta-analysis showed that high expression of PTN was significantly associated with an advanced TNM stage and a poor OS in tumor patients [48]. Similar to these studies, we also reported that PRPH was positively associated with tumor-associated macrophages.

Although RNASE7, encoding an antimicrobial peptide, was not demonstrated to be associated with CRC, the roles of itself and its family members in other cancers may indirectly verify our conclusions. Scola et al. reported that the expression of RNASE7 was gradually reduced during the malignant transformation process, showing the highest expression in healthy skin and the lowest expression in oral squamous cell carcinoma [49]. The low expression of RNase family members contributed to the loss of immune defense against bacterial infections [50–53], which is an important cause for the initiation of cancer. The knockdown of RNase L increased prostate cancer cell migration [54] and enhanced tumor growth and metastasis following implantation in the mouse prostate [55], the mechanism of which was related with an increased cell surface expression of integrin *β*1 and activation of the focal adhesion kinase-sarcoma pathway and the Ras-related C3 botulinum toxin substrate 1-guanosine triphosphatase activity [54]. Colorectal tumors with lower levels of RNase H2 exhibited a significantly shorter survival time [56]. In line with these studies, we also demonstrated that RNASE7 was downregulated in RC and patients with a higher level of RNASE7 had a longer OS compared with controls.

Some limitations should be acknowledged in this study. First, the prognostic signature panel was developed and validated based on the survival information retrospectively collected from the public datasets (TCGA and GSE56699). Prospective trials needed to be performed in our hospital to further verify the prognostic value of this signature panel. Second, the expressions of these signature DEGs were also identified using public TCGA and GSE123390 datasets. In these datasets, the number of samples in the normal group was quite smaller than that in the cancer group. This imbalance may cause a statistical problem. Consistent sample size in the RC and control groups should be designed to further confirm their expressions. Third, clinical (PCR, immunohistochemistry, and Pearson's correlation), *in vitro* (coimmunoprecipitation, knockdown, overexpression, or coincubation of immune cells), and *in vivo* (tumor transplantation, mimics, siRNA transfection, and immunotherapy) experiments should be conducted to explore the PPI relationships between our signature genes (PRPH-PTN, ASB2-CASQ2/PDK4/EPHA67, and GPR15/TCL1A-CXCL12) and assess the functions of our signature genes in the progression of RC (especially RNASE7, which was not reported in CRC previously). Fourth, other grouped variable selection methods (such as Elastic net and CoxBoost) [57] for identification of prognostic signature panels should be used individually or jointly with LASSO to identify more effective prognostic indicators for RC. Fifth, the expression, prognostic power, and functions of signature genes should be compared between the CC and RC samples.

## 5. Conclusion

Our study developed a five-mRNA signature panel (ASB2, GPR15, PRPH, RNASE7, and TCL1A) as an immune-related prognostic biomarker for RC. This signature panel exhibited excellent accuracy to stratify the patients with a higher death risk. The nomogram that combined the risk score and clinical features (age, pathologic M, pathologic N, and pathologic stage) may be more effective in guiding the clinical decision-making of personalized treatment.

## Figures and Tables

**Table 1:** Preserved modules identified based on weighted gene coexpression network analysis.

ID	Color	Module size	Preservation infor		#DEGs	Enrichment infor	
			*Z*-score	*p* value		Enrichment fold (95% CI)	*p*_hyper_
Module 1	Black	99	13.9321	5.30*e* − 11	—	—	—
Module 2	Blue	683	13.2267	1.40*e* − 30	—	—	—
Module 3	Brown	244	8.3117	4.50*e* − 19	34	2.838 (1.879-4.181)	1.38*e* − 06
Module 4	Green	226	0.9366	4.90*e* − 04	5	0.383 (0.122-0.918)	2.42*e* − 02
Module 5	Grey	2545	8.1319	6.60*e* − 32	146	1.169 (0.940-1.448)	1.55*e* − 01
Module 6	Pink	86	1.0653	1.60*e* − 02	5	1.184 (0.371-2.911)	6.17*e* − 01
Module 7	Red	123	10.5572	8.80*e* − 09	3	0.497 (0.100-1.504)	2.86*e* − 01
Module 8	Turquoise	796	8.3751	9.20*e* − 14	11	0.291 (0.143-0.533)	3.75*e* − 06
Module 9	Yellow	230	12.5199	7.10*e* − 07	43	3.807 (2.617-5.440)	2.63*e* − 11

DEGs: differentially expressed genes; CI: confidence interval.

**Table 2:** The optimal signature for prognosis prediction.

Symbol	TCGA		GSE123390		Univariate Cox		Multivariate Cox			LASSO coef
	Log_2_FC	FDR	Log_2_FC	FDR	HR	*p* value	HR	95% CI	*p* value	
ASB2	-0.53	1.11*e* − 08	-0.76	1.13*e* − 04	1.43	1.35*e* − 02	12.505	2.639-59.260	1.46*e* − 03	0.3082
GPR15	-0.77	6.48*e* − 21	-1.17	1.56*e* − 03	0.898	1.75*e* − 03	0.688	0.540-0.877	2.56*e* − 03	-0.0651
PRPH	-0.81	1.11*e* − 08	-0.58	3.94*e* − 02	1.21	4.35*e* − 02	2.048	1.030-4.069	4.09*e* − 02	0.0701
RNASE7	-0.51	1.43*e* − 04	-0.65	2.89*e* − 03	0.926	3.70*e* − 02	0.686	0.501-0.939	1.86*e* − 02	-0.1112
TCL1A	-0.98	3.73*e* − 05	-0.56	3.44*e* − 02	0.898	4.90*e* − 03	0.530	0.372-0.754	4.20*e* − 04	-0.1428

FDR: false discovery rate; FC: fold change; HR: hazard ratio; CI: confidence interval; LASSO: least absolute shrinkage and selection operator; TCGA: The Cancer Genome Atlas.

**Table 3:** Univariate and multivariate Cox regression of the clinical features and risk score.

Clinical characteristics	TCGA (*N* = 152)	Univariate Cox			Multivariate Cox		
		HR	95% CI	*p* value	HR	95% CI	*p* value
Age (years, mean ± SD)	64.16 ± 11.96	1.100	1.049-1.154	3.40*e* − 05	1.112	1.056-1.170	6.08*e* − 05
Gender (female/male)	72/80	0.762	0.321-1.807	5.36*e* − 01	—	—	—
Pathologic M (M0/M1/-)	119/21/12	3.081	1.158-8.196	1.77*e* − 02	8.911	1.065-14.591	4.36*e* − 02
Pathologic N (N0/N1/N2/-)	80/41/30/1	1.920	1.166-3.160	7.48*e* − 03	6.169	1.956-19.457	1.91*e* − 03
Pathologic T (T1/T2/T3/T4)	9/26/104/13	2.147	0.932-4.947	7.63*e* − 02	1.460	0.563-3.789	4.37*e* − 01
Pathologic stage (I/II/III/IV/-)	28/49/47/22/6	1.976	1.206-3.238	5.36*e* − 03	1.197	1.041-1.946	4.24*e* − 02
Colon polyps present (yes/no/-)	10/57/85	1.006	0.205-4.931	9.95*e* − 01	—	—	—
History of colon polyps (yes/no/-)	30/104/18	1.125	0.364-3.48	8.38*e* − 01	—	—	—
Recurrence (yes/no/-)	6/36/110	2.828	0.145-55.14	4.80*e* − 01	—	—	—
Lymphatic invasion (yes/no/-)	61/75/16	0.885	0.327-2.399	8.10*e* − 01	—	—	—
Radiotherapy (yes/no/-)	18/99/35	0.345	0.260-1.094	7.52*e* − 02	—	—	—
Risk score model status (high/low)	76/76	5.001	1.681-14.88	9.73*e* − 04	5.462	1.371-21.754	1.61*e* − 02

SD: standard deviation; HR: hazard ratio; CI: confidence interval; TCGA: The Cancer Genome Atlas.

## Data Availability

The datasets generated for this study can be found in GEO (http://www.ncbi.nlm.nih.gov/geo/; GSE123390, GSE56699) and TCGA (https://gdc-portal.nci.nih.gov/).

## Supplementary Materials

Supplementary Materials Figure S1: protein-protein interaction network constructed by crucial module genes. Red, upregulated expression; green, downregulated expression. Table S1: common DEGs identified in GSE123390 and TCGA datasets. Table S2: function enrichment for the common differentially expressed genes in two datasets. Table S3: function enrichment for genes in the PPI network. Table S4: univariate Cox regression analysis for DEGs associated with OS. Table S5: multivariate Cox regression analysis for DEGs associated with OS.

## References

[1] Paschke S., Jafarov S., Staib L. (2018). Are colon and rectal cancer two different tumor entities? A proposal to abandon the term colorectal cancer. *International Journal of Molecular Sciences*.

[2] Lee Y. C., Lee Y. L., Chuang J. P., Lee J. C. (2013). Differences in survival between colon and rectal cancer from SEER data. *PLoS One*.

[3] Ediriweera D. S., Kumarage S., Deen K. I. (2016). Comparison of hazard of death following surgery for colon versus rectal cancer. *The Ceylon Medical Journal*.

[4] Ahluwalia P., Mondal A. K., Bloomer C. (2019). Identification and clinical validation of a novel 4 gene-signature with prognostic utility in colorectal cancer. *International Journal of Molecular Sciences*.

[5] Ge W., Cai W., Bai R. (2019). A novel 4-gene prognostic signature for hypermutated colorectal cancer. *Cancer Management and Research*.

[6] Li X., Zhang Q., Zhao L. (2020). A combined four-mRNA signature associated with lymphatic metastasis for prognosis of colorectal cancer. *Journal of Cancer*.

[7] Zuo S., Dai G., Ren X. (2019). Identification of a 6-gene signature predicting prognosis for colorectal cancer. *Cancer Cell International*.

[8] Sun D., Chen J., Liu L. (2018). Establishment of a 12-gene expression signature to predict colon cancer prognosis. *PeerJ*.

[9] Chen P. F., Wang F., Zhang Z. X. (2018). A novel gene-pair signature for relapse-free survival prediction in colon cancer. *Cancer Management and Research*.

[10] Wu W., Yang Z., Long F. (2020). COL1A1 and MZB1 as the hub genes influenced the proliferation, invasion, migration and apoptosis of rectum adenocarcinoma cells by weighted correlation network analysis. *Bioorganic Chemistry*.

[11] Zhang B. D., Li Y. R., Ding L. D., Wang Y. Y., Liu H. Y., Jia B. Q. (2019). Loss of PTPN4 activates STAT3 to promote the tumor growth in rectal cancer. *Cancer Science*.

[12] Xu Z., Li Y., Cui Y., Guo Y. (2020). Identifications of candidate genes significantly associated with rectal cancer by integrated bioinformatics analysis. *Technology in Cancer Research & Treatment*.

[13] Liu B. X., Huang G. J., Cheng H. B. (2019). Comprehensive analysis of core genes and potential mechanisms in rectal cancer. *Journal of Computational Biology*.

[14] Langfelder P., Horvath S. (2008). WGCNA: an R package for weighted correlation network analysis. *BMC Bioinformatics*.

[15] Goeman J. J. (2010). L1 penalized estimation in the Cox proportional hazards model. *Biometrical Journal*.

[16] Tibshirani R. (1997). The lasso method for variable selection in the Cox model. *Statistics in Medicine*.

[17] Ritchie M. E., Phipson B., Wu D. (2015). limma powers differential expression analyses for RNA-sequencing and microarray studies. *Nucleic Acids Research*.

[18] Langfelder P., Zhang B., Horvath S. (2008). Defining clusters from a hierarchical cluster tree: the Dynamic Tree Cut package for R. *Bioinformatics*.

[19] Langfelder P., Luo R., Oldham M. C., Horvath S. (2011). Is my network module preserved and reproducible?. *PLoS Computational Biology*.

[20] Cao J., Zhang S. (2014). A Bayesian extension of the hypergeometric test for functional enrichment analysis. *Biometrics*.

[21] Szklarczyk D., Gable A. L., Lyon D. (2019). STRING v11: protein-protein association networks with increased coverage, supporting functional discovery in genome-wide experimental datasets. *Nucleic Acids Research*.

[22] Kohl M., Wiese S., Warscheid B. (2011). Cytoscape: software for visualization and analysis of biological networks. *Methods in Molecular Biology*.

[23] Chen L., Lu D., Sun K. (2019). Identification of biomarkers associated with diagnosis and prognosis of colorectal cancer patients based on integrated bioinformatics analysis. *Gene*.

[24] Mo S., Dai W., Xiang W. (2019). Prognostic and predictive value of an autophagy-related signature for early relapse in stages I-III colon cancer. *Carcinogenesis*.

[25] Zhou Z., Mo S., Dai W. (2019). Development and validation of an autophagy score signature for the prediction of post-operative survival in colorectal cancer. *Frontiers in Oncology*.

[26] Tian X., Zhu X., Yan T. (2017). Recurrence-associated gene signature optimizes recurrence-free survival prediction of colorectal cancer. *Molecular Oncology*.

[27] Xiong Y., You W., Hou M., Peng L., Zhou H., Fu Z. (2018). Nomogram integrating genomics with clinicopathologic features improves prognosis prediction for colorectal cancer. *Molecular Cancer Research*.

[28] Spinner C. A., Lamsoul I. (2019). The E3 ubiquitin ligase Asb2*α* in T helper 2 cells negatively regulates antitumor immunity in colorectal cancer. *Molecular Cancer Research*.

[29] Li H., Yan X., Liu L. (2017). T-cell leukemia/lymphoma-1A predicts the clinical outcome for patients with stage II/III colorectal cancer. *Biomedicine & Pharmacotherapy*.

[30] Wang Y., Wang X. (2019). An integrated pan-cancer analysis and structure-based virtual screening of GPR15. *International Journal of Molecular Sciences*.

[31] Zhang C., Zhou C., Wu X. J. (2014). Human CD133-positive hematopoietic progenitor cells initiate growth and metastasis of colorectal cancer cells. *Carcinogenesis*.

[32] Sakamoto A., Sugamoto Y., Tokunaga Y. (2011). Expression profiling of the ephrin (EFN) and Eph receptor (EPH) family of genes in atherosclerosis-related human cells. *The Journal of International Medical Research*.

[33] Balakrishnan A., Guruprasad K. P., Satyamoorthy K., Joshi M. B. (2018). Interleukin-6 determines protein stabilization of DNA methyltransferases and alters DNA promoter methylation of genes associated with insulin signaling and angiogenesis. *Laboratory Investigation*.

[34] Wu M., Shang X., Sun Y., Wu J., Liu G. (2020). Integrated analysis of lymphocyte infiltration-associated lncRNA for ovarian cancer via TCGA, GTEx and GEO datasets. *PeerJ*.

[35] Li S., Ma Y., Xie C. (2015). EphA6 promotes angiogenesis and prostate cancer metastasis and is associated with human prostate cancer progression. *Oncotarget*.

[36] Zhou D., Ren K., Wang J. (2018). Erythropoietin-producing hepatocellular A6 overexpression is a novel biomarker of poor prognosis in patients with breast cancer. *Oncology Letters*.

[37] Zhang C., Berndt-Paetz M., Neuhaus J. (2020). Identification of key biomarkers in bladder cancer: evidence from a bioinformatics analysis. *Diagnostics*.

[38] Wang J., Qian Y., Gao M. (2019). Overexpression of PDK4 is associated with cell proliferation, drug resistance and poor prognosis in ovarian cancer. *Cancer Management and Research*.

[39] Punt S., Corver W. E., van der Zeeuw S. A. (2015). Whole-transcriptome analysis of flow-sorted cervical cancer samples reveals that B cell expressed TCL1A is correlated with improved survival. *Oncotarget*.

[40] Shin S. J., Roh J., Cha H. J. (2015). TCL1 expression predicts overall survival in patients with mantle cell lymphoma. *European Journal of Haematology*.

[41] Samarendra H., Jones K., Petrinic T. (2017). A meta-analysis of CXCL12 expression for cancer prognosis. *British Journal of Cancer*.

[42] Stanisavljević L., Aßmus J., Storli K. E., Leh S. M., Dahl O., Myklebust M. P. (2016). CXCR4, CXCL12 and the relative CXCL12-CXCR4 expression as prognostic factors in colon cancer. *Tumour Biology*.

[43] Yu P. F., Huang Y. (2017). Downregulation of CXCL12 in mesenchymal stromal cells by TGF*β* promotes breast cancer metastasis. *Oncogene*.

[44] Cao J., Kong F. H., Liu X., Wang X. B. (2019). Immunotherapy with dendritic cells and cytokine-induced killer cells for hepatocellular carcinoma: a meta-analysis. *World Journal of Gastroenterology*.

[45] Yang T., Zhang W., Wang L. (2018). Co-culture of dendritic cells and cytokine-induced killer cells effectively suppresses liver cancer stem cell growth by inhibiting pathways in the immune system. *BMC Cancer*.

[46] Wei X., Yang S., Pu X. (2019). Tumor-associated macrophages increase the proportion of cancer stem cells in lymphoma by secreting pleiotrophin. *American Journal of Translational Research*.

[47] Huang P., Ouyang D. J., Chang S. (2018). Chemotherapy-driven increases in the CDKN1A/PTN/PTPRZ1 axis promote chemoresistance by activating the NF-*κ*B pathway in breast cancer cells. *Cell Communication and Signaling: CCS*.

[48] Zhou J., Yang Y., Zhang Y., Liu H., Dou Q. (2018). A meta-analysis on the role of pleiotrophin (PTN) as a prognostic factor in cancer. *PLoS One*.

[49] Scola N., Gambichler T., Saklaoui H. (2012). The expression of antimicrobial peptides is significantly altered in cutaneous squamous cell carcinoma and precursor lesions. *The British Journal of Dermatology*.

[50] Spencer J. D., Schwaderer A. L., Wang H. (2013). Ribonuclease 7, an antimicrobial peptide upregulated during infection, contributes to microbial defense of the human urinary tract. *Kidney International*.

[51] Kopfnagel V., Wagenknecht S., Harder J. (2018). RNase 7 strongly promotes TLR9-mediated DNA sensing by human plasmacytoid dendritic cells. *The Journal of Investigative Dermatology*.

[52] Eichler T., Bender K., Murtha M. J., Schwartz L. (2019). Ribonuclease 7 shields the kidney and bladder from invasive uropathogenic Escherichia coli infection. *Journal of the American Society of Nephrology*.

[53] Kopfnagel V., Dreyer S., Baumert K. (2020). RNase 7 promotes sensing of self-DNA by human keratinocytes and activates an antiviral immune response. *Journal of Investigative Dermatology*.

[54] Dayal S., Zhou J., Manivannan P. (2017). RNase L suppresses androgen receptor signaling, cell migration and matrix metalloproteinase activity in prostate cancer cells. *International Journal of Molecular Sciences*.

[55] Banerjee S., Li G., Li Y. (2015). RNase L is a negative regulator of cell migration. *Oncotarget*.

[56] Aden K., Bartsch K., Dahl J. (2019). Epithelial RNase H2 maintains genome integrity and prevents intestinal tumorigenesis in mice. *Gastroenterology*.

[57] Zemmour C., Bertucci F., Finetti P. (2015). Prediction of early breast cancer metastasis from DNA microarray data using high-dimensional Cox regression models. *Cancer Inform*.

