# Supplementary material for: Identification of a 5-Gene-Based Scoring System by WGCNA and LASSO to Predict Prognosis for Rectal Cancer Patients
Source: Anal Cell Pathol (Amst). 2021 Mar 23;2021:6697407. doi: 10.1155/2021/6697407 (PMC8012151; doi:10.1155/2021/6697407)
Supplement: Supplementary Materials — Figure S1: protein-protein interaction network constructed by crucial module genes. Red, upregulated expression; green, downregulated expression. Table S1: common DEGs identified in GSE123390 and TCGA datasets. Table S2: function enrichment for the common differentially expressed genes in two datasets. Table S3: function enrichment for genes in the PPI network. Table S4: univariate Cox regression analysis for DEGs associated with OS. Table S5: multivariate Cox regression analysis for DEGs associated with OS. [file 6697407.f1.docx]

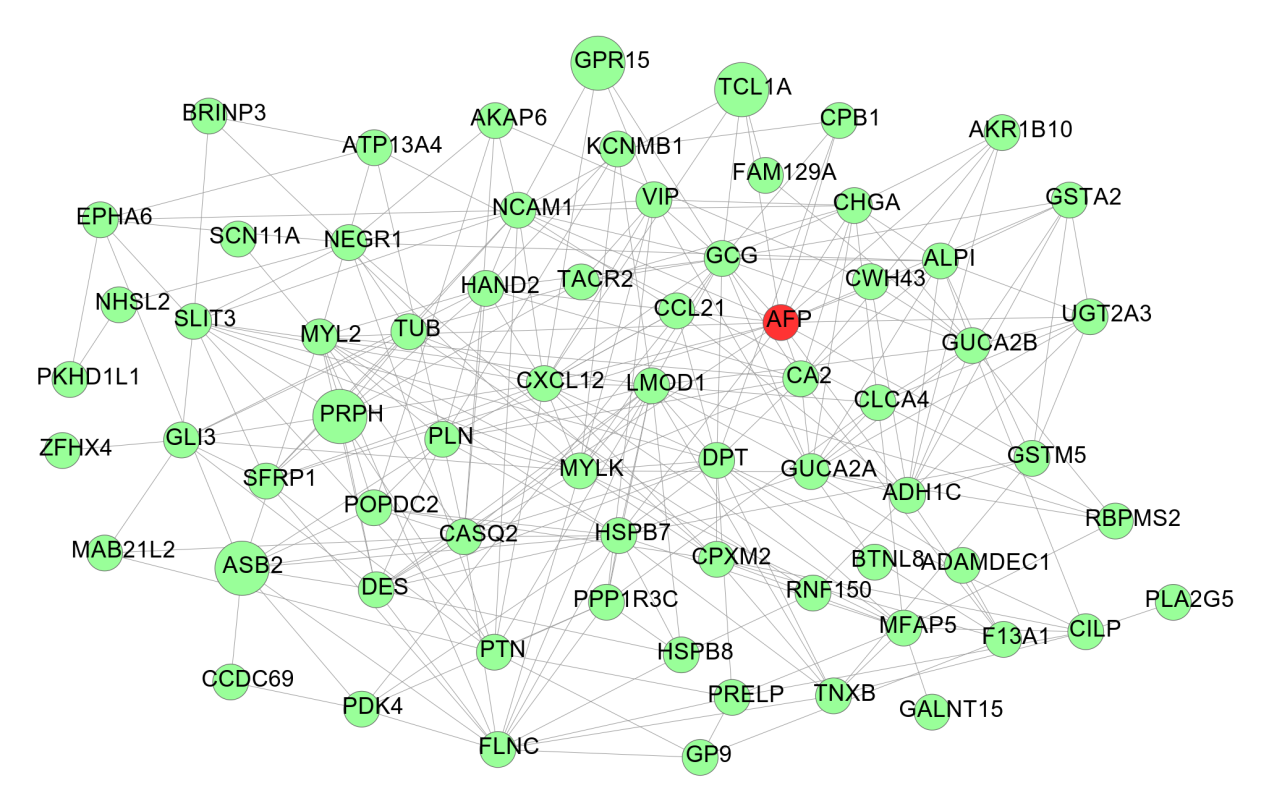


Figure S1

**Table S1 Common DEGs identified in GSE123390 and TCGA datasets**

| GSE123390 | | | |  | TCGA | | | |
| --- | --- | --- | --- | --- | --- | --- | --- | --- |
| Symbol | FDR | P-value | Log2FC |  | Symbol | P-value | FDR | Log2FC |
| DES | 7.62E-19 | 1.08E-23 | -4.74857162 |  | DES | 2.14E-07 | 1.73E-05 | -0.60217844 |
| CLCA4 | 8.14E-07 | 1.00E-09 | -2.59084807 |  | CLCA4 | 6.56E-16 | 5.31E-14 | -0.93035749 |
| MS4A12 | 2.28E-05 | 2.81E-08 | -2.23638465 |  | MS4A12 | 1.26E-15 | 1.02E-13 | -0.86678785 |
| ADH1B | 1.87E-14 | 2.31E-17 | -1.97008394 |  | ADH1B | 1.27E-23 | 1.03E-21 | -0.91472585 |
| GCG | 2.14E-03 | 2.64E-06 | -1.12034355 |  | GCG | 1.32E-26 | 1.07E-24 | -1.40120459 |
| GUCA2A | 1.57E-05 | 6.59E-08 | -2.35927712 |  | GUCA2A | 4.95E-21 | 4.01E-19 | -0.65449597 |
| UGT2B17 | 7.10E-06 | 8.77E-09 | -1.87004844 |  | UGT2B17 | 1.44E-26 | 1.17E-24 | -0.81624781 |
| HAND1 | 4.84E-05 | 5.98E-08 | -1.04889899 |  | HAND1 | 1.47E-08 | 1.19E-06 | -1.41637922 |
| FOXQ1 | 3.53E-04 | 4.36E-07 | 1.55369333 |  | FOXQ1 | 2.40E-13 | 1.94E-11 | 0.908687789 |
| FANCD2OS | 8.73E-04 | 1.08E-06 | 0.60781896 |  | FANCD2OS | 9.75E-06 | 7.89E-04 | 2.32233738 |
| MORN5 | 4.92E-09 | 6.07E-12 | -0.90315933 |  | MORN5 | 7.95E-06 | 6.44E-04 | -1.52197076 |
| MMP3 | 1.11E-02 | 7.00E-04 | 2.04633683 |  | MMP3 | 6.15E-09 | 4.98E-07 | 0.669065393 |
| SLC26A3 | 9.05E-03 | 5.15E-04 | -2.25181543 |  | SLC26A3 | 2.10E-19 | 1.70E-17 | -0.60792995 |
| INSL5 | 1.54E-03 | 1.90E-06 | -1.03304192 |  | INSL5 | 2.98E-13 | 2.41E-11 | -1.23774791 |
| SCN7A | 7.24E-12 | 8.94E-15 | -1.05662023 |  | SCN7A | 6.01E-11 | 4.87E-09 | -1.1515195 |
| PLN | 2.12E-11 | 7.50E-15 | -1.81960431 |  | PLN | 2.09E-07 | 1.69E-05 | -0.64035863 |
| SLC4A4 | 3.78E-06 | 4.67E-09 | -1.41361719 |  | SLC4A4 | 3.46E-25 | 2.80E-23 | -0.79579734 |
| ZDHHC22 | 1.56E-02 | 1.93E-05 | -0.65956541 |  | ZDHHC22 | 4.00E-09 | 3.24E-07 | -1.69733464 |
| GUCA2B | 3.36E-05 | 4.15E-08 | -1.03412181 |  | GUCA2B | 2.49E-29 | 2.02E-27 | -1.07584694 |
| LMOD1 | 6.83E-15 | 5.81E-19 | -1.92542676 |  | LMOD1 | 1.41E-07 | 1.14E-05 | -0.5765616 |
| MAB21L2 | 4.06E-12 | 1.04E-15 | -1.81337899 |  | MAB21L2 | 6.37E-07 | 5.16E-05 | -0.59456312 |
| CA2 | 1.08E-04 | 7.78E-07 | -1.625788 |  | CA2 | 4.06E-19 | 3.29E-17 | -0.65089413 |
| PGM5 | 3.97E-14 | 4.50E-18 | -1.5860961 |  | PGM5 | 4.11E-08 | 3.33E-06 | -0.65587226 |
| NRXN1 | 1.93E-05 | 2.38E-08 | -0.69704663 |  | NRXN1 | 7.99E-13 | 6.47E-11 | -1.48118853 |
| PLA2G2C | 1.12E-04 | 1.38E-07 | -0.72046895 |  | PLA2G2C | 1.20E-05 | 9.75E-04 | -1.41767756 |
| ZAR1 | 3.37E-02 | 4.16E-05 | 0.58782475 |  | ZAR1 | 7.48E-05 | 6.06E-03 | 1.730976826 |
| SST | 2.42E-04 | 2.99E-07 | -0.89164135 |  | SST | 2.51E-15 | 2.03E-13 | -1.13844476 |
| CASQ2 | 4.74E-12 | 5.85E-15 | -1.07345184 |  | CASQ2 | 4.94E-09 | 4.00E-07 | -0.93751354 |
| ASB5 | 3.71E-04 | 4.58E-07 | -0.64139073 |  | ASB5 | 2.59E-09 | 2.10E-07 | -1.56386777 |
| PRELP | 2.95E-11 | 1.17E-14 | -1.81510122 |  | PRELP | 3.41E-08 | 2.76E-06 | -0.54617071 |
| ADH1C | 1.58E-03 | 3.50E-05 | -1.57088716 |  | ADH1C | 1.08E-26 | 8.72E-25 | -0.63013988 |
| DPT | 2.86E-08 | 3.82E-11 | -1.46570306 |  | DPT | 1.52E-09 | 1.23E-07 | -0.67473549 |
| LONRF2 | 1.19E-05 | 1.47E-08 | -0.80479752 |  | LONRF2 | 3.89E-10 | 3.15E-08 | -1.2257613 |
| MAMDC2 | 1.44E-09 | 1.78E-12 | -1.11148004 |  | MAMDC2 | 1.65E-13 | 1.34E-11 | -0.88405769 |
| PDK4 | 1.61E-12 | 3.65E-16 | -1.84960287 |  | PDK4 | 1.44E-08 | 1.17E-06 | -0.52730919 |
| MYL2 | 7.35E-03 | 9.07E-06 | -0.68914323 |  | MYL2 | 4.95E-24 | 4.01E-22 | -1.41284376 |
| EPHA6 | 4.85E-03 | 5.99E-06 | -0.57272192 |  | EPHA6 | 2.17E-08 | 1.76E-06 | -1.69244159 |
| PMP2 | 3.41E-02 | 4.21E-05 | -0.54877922 |  | PMP2 | 2.85E-17 | 2.31E-15 | -1.76621698 |
| MYT1L | 3.10E-02 | 3.83E-05 | -0.5271376 |  | MYT1L | 2.90E-10 | 2.35E-08 | -1.8191104 |
| COL10A1 | 1.28E-03 | 1.58E-06 | 0.98090621 |  | COL10A1 | 2.20E-10 | 1.78E-08 | 0.976696256 |
| HS6ST3 | 3.06E-03 | 3.78E-06 | -0.66993934 |  | HS6ST3 | 2.59E-12 | 2.10E-10 | -1.4248139 |
| MGAT4C | 3.34E-02 | 4.12E-05 | -0.51524787 |  | MGAT4C | 3.35E-13 | 2.71E-11 | -1.83919396 |
| TCEAL5 | 4.96E-02 | 6.12E-05 | -0.58934321 |  | TCEAL5 | 2.19E-29 | 1.77E-27 | -1.60612806 |
| C7 | 1.03E-12 | 2.18E-16 | -1.33174319 |  | C7 | 1.42E-08 | 1.15E-06 | -0.70781149 |
| POU5F1B | 7.75E-04 | 9.57E-07 | 1.18467504 |  | POU5F1B | 9.56E-14 | 7.74E-12 | 0.780660074 |
| FLNC | 5.36E-10 | 3.04E-13 | -1.76461391 |  | FLNC | 2.79E-06 | 2.26E-04 | -0.51895719 |
| ATP1A2 | 1.47E-09 | 1.81E-12 | -0.96565234 |  | ATP1A2 | 1.67E-09 | 1.35E-07 | -0.93868933 |
| GPR15 | 1.56E-03 | 1.93E-06 | -1.17331113 |  | GPR15 | 8.00E-23 | 6.48E-21 | -0.772441 |
| RERGL | 1.08E-02 | 1.33E-05 | -0.59092691 |  | RERGL | 5.77E-15 | 4.67E-13 | -1.5283353 |
| MUSK | 1.31E-08 | 1.62E-11 | -0.77901367 |  | MUSK | 1.52E-29 | 1.23E-27 | -1.12811297 |
| CEMIP | 8.97E-03 | 5.07E-04 | 1.27767742 |  | CEMIP | 5.05E-13 | 4.09E-11 | 0.676174687 |
| RBPMS2 | 4.18E-12 | 1.18E-15 | -1.5201503 |  | RBPMS2 | 1.24E-06 | 1.00E-04 | -0.56745976 |
| CPB1 | 3.36E-04 | 4.15E-07 | -0.6135417 |  | CPB1 | 2.42E-08 | 1.96E-06 | -1.39886789 |
| BEST4 | 1.70E-09 | 2.10E-12 | -1.09911615 |  | BEST4 | 3.06E-14 | 2.48E-12 | -0.77591266 |
| PLP1 | 1.24E-05 | 1.53E-08 | -0.70547026 |  | PLP1 | 1.14E-13 | 9.20E-12 | -1.20695869 |
| ADCYAP1R1 | 2.38E-03 | 2.94E-06 | -0.66442749 |  | ADCYAP1R1 | 1.27E-10 | 1.03E-08 | -1.23948921 |
| CLDN1 | 1.02E-03 | 1.75E-05 | 1.2501627 |  | CLDN1 | 1.21E-10 | 9.77E-09 | 0.652305388 |
| SLC6A11 | 4.26E-02 | 5.26E-05 | -0.57796258 |  | SLC6A11 | 6.47E-11 | 5.24E-09 | -1.40110526 |
| FAM129A | 3.32E-10 | 1.65E-13 | -1.37130173 |  | FAM129A | 2.95E-07 | 2.39E-05 | -0.58535063 |
| HSD3B2 | 1.02E-02 | 1.26E-05 | -0.5884732 |  | HSD3B2 | 5.95E-09 | 4.82E-07 | -1.35959507 |
| AFP | 3.08E-02 | 3.80E-05 | 0.541413 |  | AFP | 1.13E-04 | 9.14E-03 | 1.476127453 |
| TMEFF2 | 1.38E-02 | 1.70E-05 | -0.56949397 |  | TMEFF2 | 4.35E-41 | 3.52E-39 | -1.38691784 |
| TNXB | 7.04E-09 | 7.58E-12 | -1.1408741 |  | TNXB | 9.74E-09 | 7.89E-07 | -0.688258 |
| CASR | 3.02E-02 | 3.73E-05 | -0.55704429 |  | CASR | 7.15E-14 | 5.79E-12 | -1.39820414 |
| RNASE3 | 7.35E-03 | 9.07E-06 | -0.87482669 |  | RNASE3 | 1.18E-08 | 9.55E-07 | -0.88285896 |
| CHRM2 | 3.13E-03 | 3.86E-06 | -0.64953735 |  | CHRM2 | 3.19E-09 | 2.58E-07 | -1.18081803 |
| CXCL5 | 1.36E-02 | 9.66E-04 | 1.34364258 |  | CXCL5 | 4.22E-05 | 3.42E-03 | 0.568031975 |
| ABCB11 | 4.40E-05 | 5.43E-08 | -0.63356532 |  | ABCB11 | 7.11E-15 | 5.76E-13 | -1.20094758 |
| INHBA | 3.78E-02 | 4.73E-03 | 1.179504 |  | INHBA | 9.10E-10 | 7.37E-08 | 0.641472645 |
| DAO | 2.66E-02 | 3.28E-05 | -0.53407121 |  | DAO | 3.96E-28 | 3.21E-26 | -1.40829528 |
| LINGO2 | 2.64E-05 | 3.26E-08 | -0.72586837 |  | LINGO2 | 8.70E-05 | 7.05E-03 | -1.0278775 |
| PCDH10 | 1.49E-02 | 1.84E-05 | -0.61950713 |  | PCDH10 | 4.09E-11 | 3.31E-09 | -1.20329539 |
| AMPD1 | 3.39E-06 | 4.19E-09 | -0.80202905 |  | AMPD1 | 4.72E-34 | 3.82E-32 | -0.92151195 |
| CYP8B1 | 6.63E-03 | 8.19E-06 | -0.72209068 |  | CYP8B1 | 2.47E-15 | 2.00E-13 | -1.02040965 |
| CHGA | 9.88E-04 | 1.22E-06 | -0.90717719 |  | CHGA | 7.75E-27 | 6.28E-25 | -0.81169059 |
| FAM135B | 8.46E-03 | 1.04E-05 | -0.55585237 |  | FAM135B | 5.19E-33 | 4.20E-31 | -1.3241686 |
| RNF150 | 3.01E-09 | 2.65E-12 | -1.05902575 |  | RNF150 | 4.74E-07 | 3.84E-05 | -0.68832398 |
| BRINP3 | 7.09E-04 | 8.75E-07 | -0.6793027 |  | BRINP3 | 7.54E-18 | 6.11E-16 | -1.06807471 |
| PTCHD1 | 5.08E-03 | 6.27E-06 | -0.71501517 |  | PTCHD1 | 8.77E-09 | 7.10E-07 | -1.00281149 |
| ALK | 2.93E-02 | 3.62E-05 | -0.56463787 |  | ALK | 1.42E-08 | 1.15E-06 | -1.26136902 |
| CNTNAP3B | 8.51E-07 | 1.05E-09 | -0.82001141 |  | CNTNAP3B | 1.38E-07 | 1.12E-05 | -0.86652947 |
| ASTN1 | 4.86E-02 | 6.00E-05 | -0.53420617 |  | ASTN1 | 1.30E-14 | 1.05E-12 | -1.32595701 |
| LRP1B | 6.17E-03 | 7.62E-06 | -0.53329285 |  | LRP1B | 9.36E-08 | 7.58E-06 | -1.30978478 |
| PRIMA1 | 6.27E-04 | 7.74E-07 | -0.71947356 |  | PRIMA1 | 8.35E-13 | 6.76E-11 | -0.95436064 |
| RD3 | 1.61E-02 | 1.99E-05 | -0.63291225 |  | RD3 | 3.87E-04 | 3.14E-02 | -1.07605258 |
| TACR2 | 5.44E-07 | 1.22E-09 | -1.03490807 |  | TACR2 | 1.95E-06 | 1.58E-04 | -0.65691652 |
| IL17F | 2.45E-02 | 3.02E-05 | 0.59039283 |  | IL17F | 3.66E-04 | 2.97E-02 | 1.147984961 |
| LMX1A | 1.78E-02 | 2.20E-05 | -0.5807209 |  | LMX1A | 6.01E-22 | 4.87E-20 | -1.15696033 |
| ADAMTSL3 | 6.22E-09 | 6.40E-12 | -0.97477718 |  | ADAMTSL3 | 4.54E-08 | 3.68E-06 | -0.68494645 |
| LYPD8 | 4.91E-03 | 1.97E-04 | -1.19598383 |  | LYPD8 | 4.60E-12 | 3.73E-10 | -0.55589629 |
| ANXA8L1 | 6.48E-03 | 8.00E-06 | -0.63084623 |  | ANXA8L1 | 3.00E-12 | 2.43E-10 | -1.04895788 |
| FMN2 | 6.64E-04 | 8.20E-07 | -0.61810231 |  | FMN2 | 1.26E-14 | 1.02E-12 | -1.07028827 |
| KCNMB1 | 3.32E-08 | 4.66E-11 | -1.11937985 |  | KCNMB1 | 4.72E-07 | 3.82E-05 | -0.59048962 |
| SFRP1 | 3.48E-05 | 4.30E-08 | -0.80427784 |  | SFRP1 | 7.27E-24 | 5.89E-22 | -0.81158841 |
| VIP | 1.45E-05 | 6.03E-08 | -1.01412905 |  | VIP | 1.10E-15 | 8.89E-14 | -0.64211513 |
| CTSG | 1.17E-05 | 1.44E-08 | -0.85464822 |  | CTSG | 1.09E-17 | 8.82E-16 | -0.75675614 |
| CCL19 | 1.21E-06 | 3.08E-09 | -1.17172149 |  | CCL19 | 5.06E-08 | 4.10E-06 | -0.55021112 |
| UGT2B10 | 1.01E-04 | 1.25E-07 | -0.73349701 |  | UGT2B10 | 8.09E-13 | 6.55E-11 | -0.87698566 |
| SLC30A10 | 1.75E-02 | 2.16E-05 | -0.60069664 |  | SLC30A10 | 4.81E-24 | 3.90E-22 | -1.06656015 |
| MFAP5 | 3.26E-07 | 6.79E-10 | -1.0396 |  | MFAP5 | 8.02E-11 | 6.50E-09 | -0.61531064 |
| CNTNAP3 | 9.24E-06 | 1.14E-08 | -0.7601813 |  | CNTNAP3 | 1.96E-10 | 1.59E-08 | -0.83594759 |
| PKHD1L1 | 1.53E-04 | 1.89E-07 | -0.59524309 |  | PKHD1L1 | 3.94E-30 | 3.19E-28 | -1.06079654 |
| GRIA1 | 3.71E-02 | 4.58E-05 | -0.53668821 |  | GRIA1 | 2.00E-09 | 1.62E-07 | -1.17416167 |
| SLC6A19 | 9.59E-03 | 1.18E-05 | -0.60257747 |  | SLC6A19 | 1.52E-25 | 1.23E-23 | -1.03208393 |
| ANO3 | 3.22E-02 | 3.98E-05 | -0.52528325 |  | ANO3 | 1.13E-12 | 9.13E-11 | -1.18148993 |
| PPP1R1A | 1.07E-04 | 1.32E-07 | -0.75368052 |  | PPP1R1A | 8.88E-07 | 7.19E-05 | -0.81951048 |
| LIPJ | 4.46E-02 | 5.51E-05 | -0.52879781 |  | LIPJ | 9.20E-14 | 7.45E-12 | -1.16763994 |
| GREM2 | 9.81E-05 | 6.81E-07 | -0.93149017 |  | GREM2 | 4.51E-19 | 3.65E-17 | -0.66123635 |
| MYLK | 9.90E-10 | 6.40E-13 | -1.11715973 |  | MYLK | 1.35E-07 | 1.09E-05 | -0.55003393 |
| HSPB8 | 3.12E-08 | 4.29E-11 | -1.03603945 |  | HSPB8 | 8.36E-07 | 6.77E-05 | -0.59240463 |
| FABP4 | 3.45E-05 | 4.26E-08 | -0.79692013 |  | FABP4 | 2.65E-09 | 2.15E-07 | -0.76680155 |
| VIT | 4.49E-05 | 5.54E-08 | -0.69589753 |  | VIT | 6.32E-15 | 5.12E-13 | -0.87522088 |
| PADI2 | 5.83E-05 | 3.56E-07 | -1.1915939 |  | PADI2 | 1.69E-13 | 1.37E-11 | -0.51071992 |
| UGT2B15 | 6.42E-03 | 3.02E-04 | -0.91494113 |  | UGT2B15 | 8.40E-14 | 6.80E-12 | -0.65155958 |
| SCN2B | 2.85E-02 | 3.52E-05 | -0.58453358 |  | SCN2B | 6.40E-12 | 5.18E-10 | -1.01793072 |
| UGT2A3 | 3.66E-03 | 1.26E-04 | -0.93003021 |  | UGT2A3 | 9.00E-23 | 7.29E-21 | -0.639404 |
| VSIG2 | 5.92E-03 | 2.62E-04 | -1.02837757 |  | VSIG2 | 6.96E-18 | 5.64E-16 | -0.57715823 |
| RSPO2 | 3.85E-04 | 4.75E-07 | -0.6208775 |  | RSPO2 | 2.67E-11 | 2.16E-09 | -0.95575388 |
| SCARA5 | 2.40E-05 | 2.96E-08 | -0.76809682 |  | SCARA5 | 6.19E-43 | 5.01E-41 | -0.75419212 |
| GP9 | 8.58E-03 | 1.06E-05 | -0.61372221 |  | GP9 | 8.96E-13 | 7.26E-11 | -0.94019578 |
| METTL24 | 8.86E-05 | 1.09E-07 | -0.7517345 |  | METTL24 | 4.02E-08 | 3.26E-06 | -0.76741397 |
| C2CD4A | 2.33E-03 | 6.21E-05 | 0.89277112 |  | C2CD4A | 1.74E-08 | 1.41E-06 | 0.646082013 |
| SEMA3D | 1.02E-05 | 1.26E-08 | -0.6734095 |  | SEMA3D | 8.30E-30 | 6.72E-28 | -0.85325098 |
| CNR1 | 6.80E-03 | 8.40E-06 | -0.61479223 |  | CNR1 | 2.93E-10 | 2.37E-08 | -0.9272997 |
| SCGN | 1.63E-02 | 2.01E-05 | -0.60246159 |  | SCGN | 2.59E-30 | 2.10E-28 | -0.946113 |
| P2RY4 | 1.28E-02 | 1.58E-05 | -0.64555947 |  | P2RY4 | 1.33E-11 | 1.08E-09 | -0.88062307 |
| CCIN | 5.16E-03 | 2.12E-04 | -0.9162783 |  | CCIN | 1.72E-07 | 1.39E-05 | -0.61344675 |
| POPDC2 | 1.14E-08 | 1.42E-11 | -0.97926681 |  | POPDC2 | 1.08E-05 | 8.72E-04 | -0.57396585 |
| AGTR1 | 5.91E-03 | 7.30E-06 | -0.59140564 |  | AGTR1 | 3.64E-10 | 2.95E-08 | -0.94972003 |
| HPGDS | 2.68E-05 | 1.28E-07 | -0.95278811 |  | HPGDS | 1.32E-10 | 1.07E-08 | -0.58717533 |
| GSTA2 | 4.66E-03 | 5.75E-06 | -0.61137093 |  | GSTA2 | 4.43E-10 | 3.59E-08 | -0.91185535 |
| CARTPT | 1.43E-02 | 1.77E-05 | -0.64283685 |  | CARTPT | 2.81E-04 | 2.27E-02 | -0.86135585 |
| GRIK3 | 1.65E-05 | 2.04E-08 | -0.73657605 |  | GRIK3 | 1.35E-07 | 1.09E-05 | -0.74942956 |
| SCN9A | 2.37E-05 | 2.93E-08 | -0.71978761 |  | SCN9A | 7.83E-22 | 6.34E-20 | -0.76670344 |
| PAPPA2 | 4.47E-05 | 5.52E-08 | -0.69228038 |  | PAPPA2 | 4.75E-09 | 3.85E-07 | -0.78791358 |
| DCT | 1.08E-02 | 1.33E-05 | -0.54724311 |  | DCT | 9.09E-08 | 7.36E-06 | -0.99564247 |
| TCL1A | 3.44E-02 | 4.25E-05 | -0.5563149 |  | TCL1A | 4.60E-07 | 3.73E-05 | -0.97754763 |
| ASCL2 | 1.43E-03 | 2.97E-05 | 0.95531587 |  | ASCL2 | 1.88E-14 | 1.52E-12 | 0.567272424 |
| ADAMDEC1 | 3.84E-02 | 4.85E-03 | -0.95011788 |  | ADAMDEC1 | 3.95E-18 | 3.20E-16 | -0.56398469 |
| KRBOX1 | 1.26E-02 | 1.56E-05 | -0.61657571 |  | KRBOX1 | 2.04E-18 | 1.65E-16 | -0.86558549 |
| KRT2 | 3.71E-02 | 4.58E-05 | -0.5521877 |  | KRT2 | 9.27E-11 | 7.51E-09 | -0.96308351 |
| PRKAA2 | 2.51E-04 | 2.39E-06 | -0.7902285 |  | PRKAA2 | 2.51E-16 | 2.03E-14 | -0.67103918 |
| CCDC69 | 4.77E-10 | 2.50E-13 | -1.01913077 |  | CCDC69 | 3.41E-07 | 2.76E-05 | -0.51106994 |
| RXRG | 1.05E-02 | 1.30E-05 | -0.56216498 |  | RXRG | 1.42E-13 | 1.15E-11 | -0.9249431 |
| ZSCAN23 | 1.56E-02 | 1.93E-05 | -0.5854753 |  | ZSCAN23 | 2.30E-15 | 1.86E-13 | -0.88580794 |
| KLF17 | 1.41E-03 | 1.74E-06 | -0.67893613 |  | KLF17 | 3.87E-04 | 3.14E-02 | -0.76294242 |
| CXCL12 | 8.78E-07 | 2.13E-09 | -1.00642736 |  | CXCL12 | 1.43E-09 | 1.16E-07 | -0.51464308 |
| NTRK3 | 1.55E-03 | 1.91E-06 | -0.6055116 |  | NTRK3 | 6.17E-17 | 5.00E-15 | -0.85454513 |
| HAND2 | 1.08E-02 | 1.33E-05 | -0.66071994 |  | HAND2 | 5.94E-08 | 4.81E-06 | -0.77338865 |
| DNAI1 | 3.51E-02 | 4.33E-05 | -0.54103532 |  | DNAI1 | 1.15E-04 | 9.28E-03 | -0.9433327 |
| NOS1 | 3.17E-02 | 3.91E-05 | -0.55220087 |  | NOS1 | 1.64E-06 | 1.33E-04 | -0.92354261 |
| CILP | 5.78E-04 | 7.87E-06 | -0.75648703 |  | CILP | 1.65E-11 | 1.34E-09 | -0.67406134 |
| SLIT3 | 3.59E-08 | 5.25E-11 | -0.96695225 |  | SLIT3 | 1.57E-08 | 1.27E-06 | -0.52303214 |
| CWH43 | 1.89E-04 | 2.33E-07 | -0.65559143 |  | CWH43 | 2.33E-25 | 1.89E-23 | -0.7635746 |
| NRAP | 1.36E-02 | 1.68E-05 | -0.56542544 |  | NRAP | 4.98E-20 | 4.03E-18 | -0.88327511 |
| PI16 | 1.81E-02 | 2.23E-05 | -0.56709952 |  | PI16 | 3.73E-13 | 3.02E-11 | -0.8776432 |
| SPTA1 | 9.90E-03 | 1.22E-05 | -0.53152323 |  | SPTA1 | 2.09E-04 | 1.70E-02 | -0.92945263 |
| SVOP | 2.45E-02 | 3.02E-05 | -0.55226635 |  | SVOP | 7.32E-15 | 5.93E-13 | -0.88621377 |
| SCN11A | 7.92E-03 | 9.78E-06 | -0.52581895 |  | SCN11A | 2.90E-14 | 2.35E-12 | -0.92342933 |
| HRASLS2 | 2.75E-03 | 8.11E-05 | -0.90372419 |  | HRASLS2 | 6.43E-10 | 5.21E-08 | -0.52606958 |
| CLEC4F | 3.20E-02 | 3.95E-05 | -0.54586357 |  | CLEC4F | 1.07E-14 | 8.69E-13 | -0.86941893 |
| PRPH | 3.94E-02 | 4.86E-05 | -0.57942125 |  | PRPH | 1.37E-10 | 1.11E-08 | -0.81072843 |
| FAM153A | 1.05E-02 | 1.30E-05 | -0.56407421 |  | FAM153A | 2.55E-04 | 2.06E-02 | -0.83028623 |
| WNT2 | 2.95E-02 | 3.64E-05 | 0.55857811 |  | WNT2 | 2.96E-09 | 2.40E-07 | 0.832077429 |
| SLC5A12 | 3.00E-02 | 3.70E-05 | -0.59809699 |  | SLC5A12 | 9.72E-17 | 7.87E-15 | -0.775284 |
| FGF10 | 1.46E-03 | 3.04E-05 | -0.6716322 |  | FGF10 | 2.88E-09 | 2.33E-07 | -0.68905818 |
| GJD4 | 4.86E-02 | 6.00E-05 | -0.58096845 |  | GJD4 | 2.95E-04 | 2.39E-02 | -0.79224406 |
| ABCC8 | 2.70E-02 | 3.33E-05 | -0.55232967 |  | ABCC8 | 3.89E-15 | 3.15E-13 | -0.83178446 |
| F13A1 | 1.85E-05 | 8.09E-08 | -0.90726623 |  | F13A1 | 2.17E-07 | 1.76E-05 | -0.50276889 |
| PITX2 | 4.56E-02 | 5.63E-05 | 0.57692059 |  | PITX2 | 2.85E-08 | 2.31E-06 | 0.781396249 |
| PGR | 3.81E-03 | 4.70E-06 | -0.59655635 |  | PGR | 7.65E-08 | 6.20E-06 | -0.75440583 |
| CCL21 | 3.92E-04 | 4.52E-06 | -0.86077616 |  | CCL21 | 8.07E-13 | 6.54E-11 | -0.52254688 |
| AKR1B10 | 2.28E-02 | 2.11E-03 | -0.80874841 |  | AKR1B10 | 5.25E-14 | 4.25E-12 | -0.55058751 |
| TRPM6 | 1.86E-02 | 1.53E-03 | -0.69963401 |  | TRPM6 | 6.02E-13 | 4.88E-11 | -0.63605134 |
| CLEC3B | 1.18E-03 | 2.20E-05 | -0.71271071 |  | CLEC3B | 1.54E-14 | 1.25E-12 | -0.6238967 |
| SLCO4C1 | 1.63E-02 | 2.01E-05 | -0.53895325 |  | SLCO4C1 | 5.22E-13 | 4.23E-11 | -0.82446074 |
| NEGR1 | 2.69E-04 | 2.63E-06 | -0.71128692 |  | NEGR1 | 4.68E-08 | 3.79E-06 | -0.62327176 |
| GSTM5 | 2.19E-05 | 9.87E-08 | -0.70275626 |  | GSTM5 | 7.95E-11 | 6.44E-09 | -0.62865942 |
| HAO2 | 1.03E-02 | 1.27E-05 | -0.58119076 |  | HAO2 | 3.82E-04 | 3.09E-02 | -0.75698081 |
| GPC5 | 2.73E-02 | 3.37E-05 | -0.57674153 |  | GPC5 | 1.62E-11 | 1.31E-09 | -0.76067137 |
| MS4A1 | 7.45E-03 | 3.79E-04 | -0.60633817 |  | MS4A1 | 2.57E-07 | 2.08E-05 | -0.71611234 |
| NCAM1 | 2.71E-06 | 7.44E-09 | -0.74978275 |  | NCAM1 | 2.20E-07 | 1.78E-05 | -0.57456449 |
| ACSM4 | 1.45E-02 | 1.79E-05 | -0.54687906 |  | ACSM4 | 1.09E-08 | 8.81E-07 | -0.77761227 |
| PTN | 6.75E-06 | 2.35E-08 | -0.79875741 |  | PTN | 3.78E-09 | 3.06E-07 | -0.52889556 |
| RYR3 | 1.09E-06 | 2.70E-09 | -0.6922529 |  | RYR3 | 3.52E-07 | 2.85E-05 | -0.60649334 |
| MEGF10 | 1.96E-02 | 2.42E-05 | -0.55984757 |  | MEGF10 | 2.20E-08 | 1.78E-06 | -0.74919386 |
| FMO2 | 5.35E-03 | 6.60E-06 | -0.56704961 |  | FMO2 | 6.40E-07 | 5.18E-05 | -0.73718686 |
| PPP1R3C | 1.28E-04 | 9.67E-07 | -0.7713225 |  | PPP1R3C | 3.04E-07 | 2.46E-05 | -0.53742423 |
| GLI3 | 5.00E-05 | 2.97E-07 | -0.8037993 |  | GLI3 | 4.59E-07 | 3.72E-05 | -0.51116873 |
| AKAP6 | 3.02E-09 | 2.70E-12 | -0.75625165 |  | AKAP6 | 2.38E-06 | 1.93E-04 | -0.54166507 |
| ASB2 | 1.13E-04 | 8.23E-07 | -0.76437664 |  | ASB2 | 1.53E-06 | 1.24E-04 | -0.53438516 |
| KY | 4.24E-02 | 5.23E-05 | -0.52385733 |  | KY | 6.14E-07 | 4.97E-05 | -0.77749838 |
| EPHX4 | 5.28E-03 | 2.19E-04 | 0.6572995 |  | EPHX4 | 1.95E-12 | 1.58E-10 | 0.612303261 |
| CPXM2 | 2.97E-06 | 8.88E-09 | -0.78814908 |  | CPXM2 | 2.74E-06 | 2.22E-04 | -0.50323855 |
| CCDC141 | 1.09E-02 | 1.35E-05 | -0.53535675 |  | CCDC141 | 4.19E-09 | 3.39E-07 | -0.73855013 |
| CNR2 | 3.84E-02 | 4.84E-03 | -0.53657602 |  | CNR2 | 1.51E-08 | 1.22E-06 | -0.73235143 |
| UBE2QL1 | 1.64E-02 | 1.29E-03 | -0.6748193 |  | UBE2QL1 | 3.93E-08 | 3.18E-06 | -0.57235603 |
| GPR27 | 7.36E-03 | 3.73E-04 | -0.64905514 |  | GPR27 | 2.21E-09 | 1.79E-07 | -0.58910724 |
| NR3C2 | 2.78E-02 | 2.90E-03 | -0.7500527 |  | NR3C2 | 6.11E-13 | 4.95E-11 | -0.50878584 |
| GFRA3 | 2.08E-02 | 1.84E-03 | -0.60626147 |  | GFRA3 | 4.12E-09 | 3.34E-07 | -0.62693146 |
| BTNL8 | 1.47E-02 | 1.08E-03 | -0.71214075 |  | BTNL8 | 2.83E-08 | 2.29E-06 | -0.52625032 |
| FAM189A2 | 6.12E-03 | 2.76E-04 | -0.61500136 |  | FAM189A2 | 6.01E-14 | 4.87E-12 | -0.60812768 |
| STOX2 | 2.27E-04 | 2.10E-06 | -0.64456439 |  | STOX2 | 2.57E-08 | 2.08E-06 | -0.57838808 |
| ALPI | 2.10E-02 | 1.86E-03 | -0.53996132 |  | ALPI | 1.85E-14 | 1.50E-12 | -0.6809027 |
| HTR4 | 7.05E-03 | 3.47E-04 | -0.51317668 |  | HTR4 | 3.95E-23 | 3.20E-21 | -0.70503563 |
| ATP13A4 | 1.38E-02 | 9.80E-04 | -0.53936661 |  | ATP13A4 | 4.28E-25 | 3.47E-23 | -0.66761703 |
| PKNOX2 | 3.83E-03 | 1.35E-04 | -0.55718017 |  | PKNOX2 | 3.52E-12 | 2.85E-10 | -0.64549008 |
| PDE7B | 1.43E-03 | 2.98E-05 | -0.65352001 |  | PDE7B | 1.04E-11 | 8.43E-10 | -0.54938827 |
| TLL1 | 1.03E-02 | 6.24E-04 | -0.52716126 |  | TLL1 | 8.60E-15 | 6.97E-13 | -0.67959809 |
| CDO1 | 2.77E-02 | 2.88E-03 | -0.55890836 |  | CDO1 | 3.79E-08 | 3.07E-06 | -0.63661341 |
| SLC51B | 1.34E-02 | 9.41E-04 | -0.66913818 |  | SLC51B | 1.73E-10 | 1.40E-08 | -0.53107441 |
| HSPB7 | 1.81E-03 | 4.23E-05 | -0.66002535 |  | HSPB7 | 1.02E-06 | 8.23E-05 | -0.53607674 |
| TRIM9 | 2.21E-03 | 5.79E-05 | -0.63818025 |  | TRIM9 | 7.42E-08 | 6.01E-06 | -0.55170393 |
| ABCD2 | 4.14E-02 | 5.46E-03 | -0.52526087 |  | ABCD2 | 2.56E-11 | 2.07E-09 | -0.66690224 |
| PRKG2 | 9.84E-03 | 5.84E-04 | -0.51920118 |  | PRKG2 | 2.37E-13 | 1.92E-11 | -0.67275245 |
| MAOB | 1.05E-05 | 4.13E-08 | -0.68160917 |  | MAOB | 2.25E-08 | 1.82E-06 | -0.51031558 |
| ADCY2 | 1.21E-03 | 2.31E-05 | -0.56398806 |  | ADCY2 | 5.31E-07 | 4.30E-05 | -0.61276923 |
| NPY4R | 9.94E-03 | 5.93E-04 | -0.57126254 |  | NPY4R | 2.26E-17 | 1.83E-15 | -0.59487102 |
| CAP2 | 1.81E-04 | 1.53E-06 | -0.65999668 |  | CAP2 | 1.45E-06 | 1.17E-04 | -0.51406106 |
| TMEM253 | 6.65E-03 | 3.19E-04 | -0.63979442 |  | TMEM253 | 1.25E-10 | 1.01E-08 | -0.52763099 |
| TRIM29 | 4.69E-02 | 6.62E-03 | 0.63276648 |  | TRIM29 | 3.93E-07 | 3.18E-05 | 0.531155332 |
| ZFHX4 | 1.62E-03 | 3.59E-05 | -0.60278655 |  | ZFHX4 | 6.22E-05 | 5.04E-03 | -0.54732057 |
| RNASE7 | 2.89E-03 | 8.72E-05 | -0.65009522 |  | RNASE7 | 1.77E-06 | 1.43E-04 | -0.50701255 |
| FIGN | 2.56E-02 | 2.53E-03 | -0.56772101 |  | FIGN | 5.58E-09 | 4.52E-07 | -0.57360547 |
| PRDM6 | 2.02E-02 | 1.76E-03 | -0.53835341 |  | PRDM6 | 1.14E-06 | 9.21E-05 | -0.60306073 |
| HOMER2 | 2.30E-03 | 6.12E-05 | -0.60205181 |  | HOMER2 | 1.91E-07 | 1.55E-05 | -0.53867312 |
| POU2AF1 | 1.65E-02 | 1.30E-03 | -0.64126148 |  | POU2AF1 | 5.65E-08 | 4.58E-06 | -0.50281455 |
| SHISA2 | 3.87E-02 | 4.91E-03 | 0.58670135 |  | SHISA2 | 3.30E-09 | 2.67E-07 | 0.536038571 |
| KLRC1 | 3.84E-02 | 4.85E-03 | -0.51199427 |  | KLRC1 | 2.74E-12 | 2.22E-10 | -0.61141203 |
| PLA2G5 | 7.13E-03 | 3.55E-04 | -0.53694354 |  | PLA2G5 | 2.26E-06 | 1.83E-04 | -0.58065481 |
| TENM2 | 2.36E-02 | 2.24E-03 | -0.51069349 |  | TENM2 | 3.32E-06 | 2.69E-04 | -0.61000729 |
| PNLIPRP2 | 2.85E-02 | 3.01E-03 | -0.50579996 |  | PNLIPRP2 | 1.80E-10 | 1.46E-08 | -0.61020296 |
| GALNT16 | 3.50E-02 | 4.16E-03 | -0.5104027 |  | GALNT16 | 1.84E-07 | 1.49E-05 | -0.59989789 |
| SPTBN2 | 8.09E-03 | 4.29E-04 | 0.58343427 |  | SPTBN2 | 1.85E-08 | 1.50E-06 | 0.52362991 |
| STAB2 | 5.83E-03 | 2.56E-04 | -0.54914275 |  | STAB2 | 2.31E-11 | 1.87E-09 | -0.55593841 |
| FAXC | 3.79E-02 | 4.75E-03 | -0.5295006 |  | FAXC | 2.57E-06 | 2.08E-04 | -0.57351086 |
| MSX2 | 3.98E-02 | 5.13E-03 | 0.54690698 |  | MSX2 | 6.73E-07 | 5.45E-05 | 0.547408496 |
| COLGALT2 | 3.49E-02 | 4.15E-03 | -0.51962128 |  | COLGALT2 | 1.46E-05 | 1.18E-03 | -0.57539304 |
| KIF5C | 1.61E-02 | 1.25E-03 | -0.50324149 |  | KIF5C | 3.88E-10 | 3.14E-08 | -0.58794653 |
| THRB | 7.05E-03 | 3.47E-04 | -0.55751011 |  | THRB | 2.94E-17 | 2.38E-15 | -0.52946618 |
| NHSL2 | 2.80E-02 | 2.93E-03 | -0.50285567 |  | NHSL2 | 1.44E-07 | 1.17E-05 | -0.58122195 |
| CR1 | 1.70E-02 | 1.35E-03 | -0.53734187 |  | CR1 | 4.41E-06 | 3.57E-04 | -0.53894311 |
| GALNT15 | 2.64E-03 | 7.58E-05 | -0.57070655 |  | GALNT15 | 9.12E-08 | 7.39E-06 | -0.5010427 |
| MPZ | 4.13E-02 | 5.44E-03 | -0.50743991 |  | MPZ | 1.26E-07 | 1.02E-05 | -0.56039837 |
| TUB | 2.81E-02 | 2.94E-03 | -0.53608018 |  | TUB | 1.58E-07 | 1.28E-05 | -0.52176988 |
| DOCK3 | 2.05E-03 | 5.13E-05 | -0.54175359 |  | DOCK3 | 4.28E-05 | 3.47E-03 | -0.50712179 |
| CACNA2D1 | 2.29E-02 | 2.13E-03 | -0.51346299 |  | CACNA2D1 | 1.59E-06 | 1.29E-04 | -0.525377 |
| MAP9 | 2.31E-02 | 2.17E-03 | 0.57291419 |  | MAP9 | 2.26E-08 | 1.83E-06 | -0.52472434 |
| LRRTM2 | 1.34E-02 | 9.43E-04 | 0.69422711 |  | LRRTM2 | 3.40E-08 | 2.75E-06 | -0.50594953 |
| FAM181B | 3.22E-02 | 3.98E-05 | 0.62570827 |  | FAM181B | 6.35E-10 | 5.14E-08 | -0.77725378 |
| HTR2B | 8.89E-03 | 4.98E-04 | 0.7526318 |  | HTR2B | 1.70E-08 | 1.38E-06 | -0.6535901 |
| RAET1L | 9.80E-03 | 1.21E-05 | -0.58645593 |  | RAET1L | 4.21E-04 | 3.41E-02 | 0.881939505 |
| C1QTNF9 | 1.62E-02 | 2.00E-05 | 0.57828154 |  | C1QTNF9 | 1.69E-16 | 1.37E-14 | -0.9214639 |
| GCKR | 4.24E-02 | 5.23E-05 | -0.53878846 |  | GCKR | 3.21E-04 | 2.60E-02 | 1.027645047 |
| MUCL1 | 4.31E-02 | 5.32E-05 | -0.5641003 |  | MUCL1 | 4.81E-04 | 3.90E-02 | 1.127518665 |
| NR0B1 | 1.99E-02 | 2.46E-05 | 0.57540413 |  | NR0B1 | 3.49E-05 | 2.83E-03 | -1.15362185 |
| AHSG | 4.94E-04 | 6.10E-07 | -0.68795875 |  | AHSG | 4.94E-04 | 4.00E-02 | 0.976504994 |
| GPR22 | 1.16E-03 | 1.43E-06 | 0.71092513 |  | GPR22 | 5.53E-05 | 4.48E-03 | -1.08928965 |
| DMRTA1 | 1.30E-04 | 1.60E-07 | 0.8851111 |  | DMRTA1 | 1.80E-22 | 1.46E-20 | -0.98715941 |
| C9orf57 | 9.30E-03 | 1.15E-05 | -0.60068917 |  | C9orf57 | 3.54E-04 | 2.87E-02 | 1.469685556 |
| SMIM18 | 6.04E-03 | 7.46E-06 | 0.69043601 |  | SMIM18 | 1.52E-05 | 1.23E-03 | -1.28254979 |
| ASCL1 | 4.71E-02 | 5.81E-05 | 0.62171054 |  | ASCL1 | 4.74E-16 | 3.84E-14 | -1.43270175 |
| ATP6V0A4 | 3.55E-02 | 4.38E-05 | -0.53179979 |  | ATP6V0A4 | 2.30E-05 | 1.86E-03 | 1.905217014 |
| CALHM1 | 9.86E-04 | 1.22E-06 | 1.12092103 |  | CALHM1 | 8.38E-17 | 6.79E-15 | -1.1136502 |
| SPZ1 | 8.22E-03 | 1.01E-05 | -0.70303284 |  | SPZ1 | 5.65E-05 | 4.58E-03 | 2.039498023 |
| DIRC1 | 3.42E-02 | 4.22E-05 | -0.56888453 |  | DIRC1 | 1.93E-06 | 1.56E-04 | 2.989529755 |
| PRR9 | 3.37E-03 | 4.16E-06 | -0.84706596 |  | PRR9 | 1.78E-05 | 1.44E-03 | 2.219523297 |
| VGLL1 | 1.13E-02 | 1.40E-05 | -0.61582521 |  | VGLL1 | 7.72E-08 | 6.25E-06 | 3.344120703 |

**Table S2 Function enrichment for the common differentially expressed genes in two datasets**

| Source | Term_Name | Term_ID | FDR | Intersections |
| --- | --- | --- | --- | --- |
| GO:MF | glycosaminoglycan binding | GO:0005539 | 2.66E-04 | PRELP, CEMIP, TNXB, SFRP1, CTSG, GREM2, VIT, RSPO2, SLIT3, FGF10, CLEC3B, PTN, RNASE7, PLA2G5, STAB2 |
| GO:MF | sodium ion transmembrane transporter activity | GO:0015081 | 2.87E-03 | SCN7A, SLC4A4, ATP1A2, SLC6A11, ABCB11, SLC6A19, SCN2B, GRIK3, SCN9A, SCN11A, SLC5A12 |
| GO:MF | metal ion transmembrane transporter activity | GO:0046873 | 3.34E-03 | SCN7A, SLC4A4, ATP1A2, SLC6A11, ABCB11, KCNMB1, SLC30A10, SLC6A19, SCN2B, ABCC8… |
| GO:MF | heparin binding | GO:0008201 | 7.28E-03 | PRELP, TNXB, SFRP1, CTSG, GREM2, RSPO2, SLIT3, FGF10, CLEC3B, PTN, PLA2G5 |
| GO:MF | adenylate cyclase binding | GO:0008179 | 7.93E-03 | ADCYAP1R1, AKAP6, ADCY2, CAP2 |
| GO:MF | inorganic cation transmembrane transporter activity | GO:0022890 | 2.60E-02 | SCN7A, SLC4A4, ATP1A2, SLC6A11, ABCB11, KCNMB1, SLC30A10, GRIA1, SLC6A19, SCN2B… |
| GO:MF | receptor ligand activity | GO:0048018 | 3.60E-02 | GCG, GUCA2A, INSL5, SST, CXCL5, INHBA, IL17F, VIP, CCL19, GREM2, SEMA3D, CARTPT, CXCL12, WNT2, FGF10, CCL21, PTN, C1QTNF9 |
| GO:MF | signaling receptor activator activity | GO:0030546 | 4.21E-02 | GCG, GUCA2A, INSL5, SST, CXCL5, INHBA, IL17F, VIP, CCL19, GREM2, SEMA3D, CARTPT, CXCL12, WNT2, FGF10, CCL21, PTN, C1QTNF9 |
| GO:MF | inorganic molecular entity transmembrane transporter activity | GO:0015318 | 4.61E-02 | CLCA4, SLC26A3, SCN7A, SLC4A4, ATP1A2, BEST4, SLC6A11, ABCB11, KCNMB1, SLC30A10… |
| GO:BP | response to endogenous stimulus | GO:0009719 | 1.5E-06 | GCG, SLC26A3, PLN, CA2, NRXN1, SST, CASQ2, PDK4, CXCL12, TUB… |
| GO:BP | response to lipid | GO:0033993 | 2.53E-06 | PLN, CA2, SST, PDK4, ATP1A2, ADCYAP1R1, CLDN1, HSD3B2, SLIT3, PTN… |
| GO:BP | ion transport | GO:0006811 | 5.68E-06 | CLCA4, GCG, SLC26A3, PLN, SLC4A4, CA2, CASQ2, TACR2, VIP, CXCL12… |
| GO:BP | response to oxygen-containing compound | GO:1901700 | 9.12E-06 | GCG, MMP3, SLC26A3, PLN, CA2, SST, PDK4, ATP1A2, CXCL12, SLIT3… |
| GO:BP | response to hormone | GO:0009725 | 1.72E-05 | GCG, PLN, CA2, SST, PDK4, ATP1A2, ADCYAP1R1, CLDN1, HSD3B2, CHRM2, INHBA, SFRP1, CXCL12, SLIT3, TUB… |
| GO:BP | response to organic cyclic compound | GO:0014070 | 2.77E-05 | SLC26A3, PLN, CA2, SST, CASQ2, ATP1A2, ADCYAP1R1, CLDN1, KCNMB1, SLIT3… |
| GO:BP | blood circulation | GO:0008015 | 1.21E-04 | DES, PLN, GUCA2B, CASQ2, MYL2, ATP1A2, TACR2, VIP, POPDC2, CXCL12… |
| GO:BP | cellular chemical homeostasis | GO:0055082 | 1.55E-04 | GCG, SLC26A3, PLN, SLC4A4, CA2, CASQ2, C7, ATP1A2, CEMIP, CXCL12… |
| GO:BP | circulatory system process | GO:0003013 | 1.72E-04 | DES, PLN, GUCA2B, CASQ2, MYL2, ATP1A2, CASR, TACR2, VIP, CXCL12… |
| GO:BP | behavior | GO:0007610 | 2.66E-04 | GCG, INSL5, NRXN1, ATP1A2, MUSK, CASR, PTCHD1, TACR2, VIP, CXCL12… |
| GO:BP | regulation of system process | GO:0044057 | 3.45E-04 | DES, PLN, NRXN1, CASQ2, MYL2, ATP1A2, CASR, CHRM2, INHBA, CHGA, TACR2, VIP, SCN2B, POPDC2, CARTPT… |
| GO:BP | regulation of ion transport | GO:0043269 | 4.191E-04 | GCG, SCN7A, PLN, CA2, NRXN1, CASQ2, ATP1A2, TACR2, VIP, CXCL12… |
| GO:BP | chemical homeostasis | GO:0048878 | 6.80E-04 | GCG, SLC26A3, SCN7A, PLN, SLC4A4, CA2, CASQ2, PDK4, C7, CXCL12… |
| GO:BP | response to alcohol | GO:0097305 | 8.54E-04 | ADCYAP1R1, CLDN1, HSD3B2, INHBA, KCNMB1, SFRP1, CCL19, CNR1, P2RY4, PRKAA2, NTRK3, SLIT3, CCL21, CDO1, MAOB, ADCY2 |
| GO:BP | cellular response to endogenous stimulus | GO:0071495 | 8.58E-04 | GCG, SLC26A3, CA2, NRXN1, SST, CASQ2, PDK4, ATP1A2, ALK, SLIT3… |
| GO:BP | metal ion transport | GO:0030001 | 1.14E-03 | GCG, SCN7A, PLN, SLC4A4, CASQ2, ATP1A2, CEMIP, VIP, MYLK, CXCL12… |
| GO:BP | ion homeostasis | GO:0050801 | 1.20E-03 | SLC26A3, SCN7A, PLN, SLC4A4, CA2, CASQ2, PDK4, C7, GRIA1, CXCL12… |
| GO:BP | cation transport | GO:0006812 | 1.25E-03 | GCG, SCN7A, PLN, SLC4A4, NRXN1, CASQ2, ATP1A2, TACR2, VIP, CXCL12… |
| GO:BP | cation homeostasis | GO:0055080 | 1.30E-03 | SLC26A3, SCN7A, PLN, SLC4A4, CA2, CASQ2, PDK4, C7, ATP1A2, CXCL12… |
| GO:BP | cellular cation homeostasis | GO:0030003 | 1.66E-03 | SLC26A3, PLN, SLC4A4, CA2, CASQ2, C7, ATP1A2, CEMIP, ADCYAP1R1, CXCL12… |
| GO:BP | calcium ion transport into cytosol | GO:0060402 | 1.74E-03 | PLN, CASQ2, ATP1A2, CEMIP, ADCYAP1R1, CCL19, NOS1, CCL21, MS4A1, RYR3, AKAP6, CACNA2D1, HTR2B |
| GO:BP | inorganic ion homeostasis | GO:0098771 | 1.79E-03 | SLC26A3, SCN7A, PLN, SLC4A4, CA2, CASQ2, PDK4, C7, GRIA1, CXCL12… |
| GO:BP | cellular ion homeostasis | GO:0006873 | 2.46E-03 | SLC26A3, PLN, SLC4A4, CA2, CASQ2, C7, ATP1A2, CEMIP, CNR1, CXCL12… |
| GO:BP | cellular homeostasis | GO:0019725 | 4.18E-03 | GCG, SLC26A3, PLN, SLC4A4, CA2, CASQ2, C7, ATP1A2, CNR1, CXCL12… |
| GO:BP | cytosolic calcium ion transport | GO:0060401 | 5.64E-03 | PLN, CASQ2, ATP1A2, CEMIP, ADCYAP1R1, CCL19, NOS1, CCL21, MS4A1, RYR3, AKAP6, CACNA2D1, HTR2B |
| GO:BP | cellular calcium ion homeostasis | GO:0006874 | 6.42E-03 | PLN, CASQ2, ATP1A2, CEMIP, ADCYAP1R1, CASR, CCL19, GRIA1, CNR1, SCGN, P2RY4… |
| GO:BP | response to drug | GO:0042493 | 6.45E-03 | MMP3, CA2, SST, CASQ2, ATP1A2, ADCYAP1R1, CLDN1, SLC6A11, TACR2, CXCL12… |
| GO:BP | response to organic substance | GO:0010033 | 8.31E-03 | GCG, MMP3, SLC26A3, PLN, CA2, NRXN1, CASQ2, PDK4, CXCL12, TUB… |
| GO:BP | calcium ion homeostasis | GO:0055074 | 9.46E-03 | PLN, CASQ2, ATP1A2, CEMIP, ADCYAP1R1, CASR, CCL19, GRIA1, CXCL12, NOS1… |
| GO:BP | regulation of cytosolic calcium ion concentration | GO:0051480 | 9.72E-03 | PLN, CASQ2, ATP1A2, CEMIP, ADCYAP1R1, CCL19, GRIA1, CNR1, CCL21, MS4A1… |
| GO:BP | regulation of blood circulation | GO:1903522 | 1.07E-02 | DES, PLN, CASQ2, MYL2, ATP1A2, CASR, CHRM2, CHGA, SCN2B, POPDC2, AGTR1, NOS1, YR3, HSPB7, THRB, CACNA2D1 |
| GO:BP | response to ketone | GO:1901654 | 1.10E-02 | PLN, CLDN1, HSD3B2, SFRP1, CCL19, P2RY4, PRKAA2, NTRK3, SLIT3, CCL21, PTN, MAOB, ADCY2 |
| GO:BP | calcium ion transport | GO:0006816 | 1.17E-02 | GCG, PLN, CASQ2, ATP1A2, CEMIP, ADCYAP1R1, CASR, CCL19, MYLK, CXCL12… |
| GO:BP | response to chemical | GO:0042221 | 1.22E-02 | GCG, MMP3, SLC26A3, PLN, EPHA6, CASQ2, PDK4, TACR2, MYLK, TCL1A, CXCL12, TUB… |
| GO:BP | divalent metal ion transport | GO:0070838 | 1.42E-02 | GCG, PLN, CASQ2, ATP1A2, CEMIP, ADCYAP1R1, CASR, CCL19, MYLK, CXCL12… |
| GO:BP | ion transmembrane transport | GO:0034220 | 1.47E-02 | CLCA4, SLC26A3, SCN7A, PLN, SLC4A4, NRXN1, CASQ2, ATP1A2, CCL19, GRIA1… |
| GO:BP | divalent inorganic cation transport | GO:0072511 | 1.75E-02 | GCG, PLN, CASQ2, ATP1A2, CEMIP, ADCYAP1R1, CASR, CCL19, MYLK, CXCL12… |
| GO:BP | cellular divalent inorganic cation homeostasis | GO:0072503 | 1.86E-02 | PLN, CASQ2, ATP1A2, CEMIP, ADCYAP1R1, CASR, CCL19, GRIA1, CXCL12, NOS1… |
| GO:BP | calcium ion transmembrane import into cytosol | GO:0097553 | 1.87E-02 | PLN, CASQ2, ATP1A2, CEMIP, CCL19, NOS1, CCL21, RYR3, AKAP6, CACNA2D1, HTR2B |
| GO:BP | membrane depolarization during action potential | GO:0086010 | 1.90E-02 | SCN7A, ATP1A2, SCN2B, SCN9A, SCN11A, CACNA2D1 |
| GO:BP | cellular metal ion homeostasis | GO:0006875 | 2.21E-02 | PLN, CASQ2, C7, ATP1A2, CEMIP, ADCYAP1R1, CASR, CCL19, GRIA1, CXCL12… |
| GO:BP | chemotaxis | GO:0006935 | 2.64E-02 | NRXN1, EPHA6, TMEFF2, CASR, CXCL5, CHGA, LMX1A, CCL19, CXCL12, SLIT3… |
| GO:BP | regulation of heart contraction | GO:0008016 | 2.69E-02 | DES, PLN, CASQ2, MYL2, ATP1A2, CHRM2, CHGA, SCN2B, POPDC2, NOS1, RYR3, HSPB7, THRB, CACNA2D1 |
| GO:BP | taxis | GO:0042330 | 2.77E-02 | NRXN1, EPHA6, TMEFF2, CASR, CXCL5, CHGA, LMX1A, CXCL12, SLIT3, PTN… |
| GO:BP | regulation of membrane potential | GO:0042391 | 2.85E-02 | SLC26A3, SCN7A, PLN, NRXN1, CASQ2, ATP1A2, GRIA1, SCN2B, POPDC2, PTN… |
| GO:BP | divalent inorganic cation homeostasis | GO:0072507 | 3.23E-02 | PLN, CASQ2, ATP1A2, CEMIP, ADCYAP1R1, CASR, CCL19, GRIA1, CXCL12, NOS1… |
| GO:BP | cell-cell signaling | GO:0007267 | 3.35E-02 | GCG, CA2, NRXN1, SST, CASQ2, ATP1A2, PLP1, CASR, CHRM2, CXCL5, INHBA, CHGA, TACR2, VIP, PTN… |
| GO:BP | regulation of metal ion transport | GO:0010959 | 3.53E-02 | GCG, PLN, CASQ2, ATP1A2, CEMIP, ADCYAP1R1, CASR, VIP, MYLK, CXCL12… |
| GO:BP | action potential | GO:0001508 | 3.66E-02 | SCN7A, ATP1A2, SCN2B, CNR1, SCN9A, NTRK3, SCN11A, AKAP6, CNR2, CACNA2D1 |
| GO:BP | response to organonitrogen compound | GO:0010243 | 0.036762 | GCG, MMP3, SLC26A3, PLN, CA2, SST, CASQ2, PDK4, CASR, CHRM2, ALK, SFRP1, SLC30A10, CNR1, CXCL12… |
| GO:BP | positive regulation of cytosolic calcium ion concentration | GO:0007204 | 3.75E-02 | PLN, CASQ2, ATP1A2, CEMIP, ADCYAP1R1, CCL19, CNR1, P2RY4, AGTR1, NOS1, CCL21, MS4A1, RYR3, AKAP6, CACNA2D1, HTR2B |
| GO:BP | release of sequestered calcium ion into cytosol | GO:0051209 | 4.17E-02 | PLN, CASQ2, ATP1A2, CEMIP, CCL19, NOS1, CCL21, RYR3, AKAP6, HTR2B |
| GO:BP | cellular response to lipid | GO:0071396 | 4.24E-02 | PDK4, ATP1A2, CLDN1, CASR, CXCL5, INHBA, BRINP3, KCNMB1, RXRG, NTRK3… |
| GO:BP | cellular response to organic cyclic compound | GO:0071407 | 4.24E-02 | SLC26A3, CASQ2, ATP1A2, CASR, CHRM2, INHBA, ALK, KCNMB1, SFRP1, PADI2, P2RY4, RXRG, PGR, PTN, RYR3… |
| GO:BP | response to prostaglandin E | GO:0034695 | 4.32E-02 | SFRP1, CCL19, P2RY4, PRKAA2, CCL21 |
| GO:BP | metal ion homeostasis | GO:0055065 | 4.54E-02 | SCN7A, PLN, CASQ2, C7, ATP1A2, CEMIP, ADCYAP1R1, CASR, CCL19, CXCL12… |
| GO:BP | negative regulation of sequestering of calcium ion | GO:0051283 | 4.73E-02 | PLN, CASQ2, ATP1A2, CEMIP, CCL19, NOS1, CCL21, RYR3, AKAP6, HTR2B |
| KEGG | Drug metabolism - cytochrome P450 | KEGG:00982 | 1.67E-06 | ADH1B, UGT2B17, ADH1C, UGT2B10, UGT2B15, UGT2A3, HPGDS, GSTA2, GSTM5, FMO2, MAOB |
| KEGG | Metabolism of xenobiotics by cytochrome P450 | KEGG:00980 | 3.24E-04 | ADH1B, UGT2B17, ADH1C, UGT2B10, UGT2B15, UGT2A3, HPGDS, GSTA2, GSTM5 |
| KEGG | Chemical carcinogenesis | KEGG:05204 | 7.00E-04 | ADH1B, UGT2B17, ADH1C, UGT2B10, UGT2B15, UGT2A3, HPGDS, GSTA2, GSTM5 |
| KEGG | Pancreatic secretion | KEGG:04972 | 7.54E-04 | CLCA4, SLC26A3, SLC4A4, CA2, PLA2G2C, ATP1A2, CPB1, ADCY2, PLA2G5, PNLIPRP2 |
| KEGG | Neuroactive ligand-receptor interaction | KEGG:04080 | 1.41E-03 | GCG, INSL5, SST, ADCYAP1R1, CHRM2, TACR2, VIP, CTSG, GRIA1, HTR4… |
| KEGG | Pentose and glucuronate interconversions | KEGG:00040 | 2.25E-02 | UGT2B17, UGT2B10, UGT2B15, UGT2A3, AKR1B10 |
| KEGG | Calcium signaling pathway | KEGG:04020 | 3.77E-02 | PLN, CASQ2, CHRM2, TACR2, MYLK, AGTR1, NOS1, RYR3, HTR4, ADCY2, HTR2B |
| REAC | Muscle contraction | REAC:R-HSA-397014 | 1.55E-03 | DES, SCN7A, PLN, LMOD1, CASQ2, MYL2, ATP1A2, MYLK, SCN2B, SCN9A, NOS1, SCN11A, RYR3, CACNA2D1 |
| REAC | Interaction between L1 and Ankyrins | REAC:R-HSA-445095 | 2.73E-03 | SCN7A, SCN2B, SCN9A, SPTA1, SCN11A, SPTBN2 |
| REAC | GPCR ligand binding | REAC:R-HSA-500792 | 1.12E-02 | GCG, INSL5, SST, ADCYAP1R1, CASR, CHRM2, CXCL5, TACR2, VIP, CXCL12… |
| REAC | Biological oxidations | REAC:R-HSA-211859 | 1.34E-02 | ADH1B, UGT2B17, ADH1C, CYP8B1, UGT2B10, UGT2B15, UGT2A3, HPGDS, GSTA2, GSTM5, ACSM4, FMO2, MAOB |
| REAC | Cardiac conduction | REAC:R-HSA-5576891 | 2.34E-02 | SCN7A, PLN, CASQ2, ATP1A2, SCN2B, SCN9A, NOS1, SCN11A, RYR3, CACNA2D1 |

FDR, false discovery rate; GO, Gene Ontology; BP, biological process; MF, molecular function; KEGG, Kyoto Encyclopedia of Genes and Genomes; REAC, Reactome. Top 10 enriched genes were listed for some results with large gene number.

**Table S3 Function enrichment for genes in the PPI network**

|  | Term ID | Term description | FDR | Matching proteins |
| --- | --- | --- | --- | --- |
| GO:BP | GO:0044057 | regulation of system process | 1.20E-02 | AKAP6, CASQ2, CHGA, DES, HAND2, HSPB7, MYL2, PLN, POPDC2, SCN11A, TACR2 |
| GO:BP | GO:0003012 | muscle system process | 1.72E-02 | CASQ2, CHGA, DES, LMOD1, MYL2, MYLK, PLN, TACR2 |
| GO:BP | GO:0008016 | regulation of heart contraction | 1.72E-02 | CASQ2, CHGA, DES, HSPB7, MYL2, PLN, POPDC2 |
| GO:BP | GO:0090257 | regulation of muscle system process | 1.72E-02 | AKAP6, CASQ2, CHGA, HAND2, MYL2, PLN, TACR2 |
| GO:BP | GO:0019933 | cAMP-mediated signaling | 2.66E-02 | AKAP6, CHGA,GCG, PLN,VIP |
| GO:BP | GO:0008015 | blood circulation | 3.16E-02 | CASQ2, CHGA, CXCL12, GUCA2B, KCNMB1, MYL2, PLN, TACR2 |
| GO:BP | GO:0043269 | regulation of ion transport | 3.17E-02 | AKAP6, CA2, CASQ2, CXCL12, GCG, KCNMB1, MYLK, PLN, SCN11A, TACR2 |
| GO:BP | GO:2001260 | regulation of semaphorin-plexin signaling pathway | 3.17E-02 | HAND2, NCAM1 |
| GO:BP | GO:0002026 | regulation of the force of heart contraction | 3.78E-02 | CHGA, MYL2, PLN |
| GO:BP | GO:0010880 | regulation of release of sequestered calcium ion into cytosol by sarcoplasmic reticulum | 3.78E-02 | AKAP6, CASQ2, PLN |
| GO:BP | GO:0019730 | antimicrobial humoral response | 3.78E-02 | CCL21, CHGA, CXCL12, GPR15, RNASE7, VIP |
| GO:BP | GO:0034103 | regulation of tissue remodeling | 3.78E-02 | CA2, HAND2, PDK4, SFRP1 |
| GO:BP | GO:0051924 | regulation of calcium ion transport | 3.78E-02 | AKAP6, ASB2, CASQ2, CXCL12, GCG, MYLK, PLN |
| GO:BP | GO:0060314 | regulation of ryanodine-sensitive calcium-release channel activity | 3.78E-02 | AKAP6, CASQ2, PLN, TCL1A |
| GO:BP | GO:0070459 | prolactin secretion | 3.78E-02 | TACR2, VIP |
| GO:BP | GO:0086023 | adenylate cyclase-activating adrenergic receptor signaling pathway involved in heart process | 3.78E-02 | CHGA, PLN |
| GO:BP | GO:1903237 | negative regulation of leukocyte tethering or rolling | 3.78E-02 | CCL21, CXCL12 |
| GO:BP | GO:0006936 | muscle contraction | 3.87E-02 | CASQ2, DES, LMOD1, MYL2, MYLK, TACR2 |
| GO:BP | GO:0006937 | regulation of muscle contraction | 3.87E-02 | CASQ2, CHGA, MYL2, PLN, TACR2 |
| GO:BP | GO:0006942 | regulation of striated muscle contraction | 3.87E-02 | CASQ2, CHGA, MYL2, PLN |
| GO:BP | GO:0007189 | adenylate cyclase-activating G protein-coupled receptor signaling pathway | 3.87E-02 | CHGA, GCG, PLN, VIP |
| GO:BP | GO:0090279 | regulation of calcium ion import | 3.87E-02 | CXCL12, GCG, PLN |
| GO:BP | GO:0043270 | positive regulation of ion transport | 3.94E-02 | AKAP6, CXCL12, GCG, KCNMB1, MYLK, TACR2 |
| GO:BP | GO:0003015 | heart process | 4.04E-02 | CASQ2,CHGA,MYL2,PLN |
| GO:BP | GO:0010959 | regulation of metal ion transport | 4.04E-02 | AKAP6, CASQ2, CXCL12, GCG, KCNMB1, MYLK, PLN |
| GO:BP | GO:1901897 | regulation of relaxation of cardiac muscle | 4.04E-02 | CHGA, PLN |
| GO:BP | GO:2000669 | negative regulation of dendritic cell apoptotic process | 4.04E-02 | CCL21, CXCL12 |
| GO:BP | GO:0019932 | second-messenger-mediated signaling | 4.25E-02 | AKAP6, CASQ2, CHGA, GCG, PLN, VIP |
| GO:BP | GO:0097435 | supramolecular fiber organization | 4.67E-02 | CASQ2, DES, DPT, LMOD1, MFAP5, MYL2, TNXB |
| GO:BP | GO:1901654 | response to ketone | 4.75E-02 | CCL21, PLN, PTN, SFRP1, SLIT3 |
| KEGG | hsa00980 | Metabolism of xenobiotics by cytochrome P450 | 1.34E-02 | ADH1C, GSTA2, GSTM5, UGT2A3 |
| KEGG | hsa00982 | Drug metabolism - cytochrome P450 | 1.34E-02 | ADH1C, GSTA2, GSTM5, UGT2A3 |
| KEGG | hsa04972 | Pancreatic secretion | 1.34E-02 | CA2, CLCA4, CPB1, PLA2G5 |
| KEGG | hsa05204 | Chemical carcinogenesis | 1.34E-02 | ADH1C, GSTA2, GSTM5, UGT2A3 |
| REAC | HSA-397014 | Muscle contraction | 2.50E-03 | CASQ2, DES, LMOD1, MYL2, MYLK, PLN, SCN11A |
| REAC | HSA-8935690 | Digestion | 8.20E-03 | ALPI, GUCA2A, GUCA2B |
| REAC | HSA-8963743 | Digestion and absorption | 8.90E-03 | ALPI, GUCA2A, GUCA2B |
| REAC | HSA-156580 | Phase II - Conjugation of compounds | 3.48E-02 | ACSM4, GSTA2, GSTM5, UGT2A3 |

FDR, false discovery rate; GO, Gene Ontology; BP, biological process; KEGG, Kyoto Encyclopedia of Genes and Genomes; REAC, Reactome.

**Table S4 Univariate Cox regression analysis for DEGs associated with OS**

| Gene | Coef | Exp(coef) | Se(coef) | Z | P-value |
| --- | --- | --- | --- | --- | --- |
| GPR15 | -0.108 | 0.898 | 0.037 | -2.92 | 0.00175 |
| AKR1B10 | -0.145 | 0.865 | 0.0511 | -2.83 | 0.00235 |
| MYL2 | 0.118 | 1.13 | 0.0455 | 2.6 | 0.0047 |
| TCL1A | -0.107 | 0.898 | 0.0416 | -2.58 | 0.0049 |
| RBPMS2 | 0.337 | 1.4 | 0.14 | 2.41 | 0.008 |
| FAM129A | 0.423 | 1.53 | 0.179 | 2.36 | 0.009 |
| EPHX4 | -0.298 | 0.742 | 0.129 | -2.31 | 0.0105 |
| ASB2 | 0.357 | 1.43 | 0.161 | 2.21 | 0.0135 |
| POPDC2 | 0.401 | 1.49 | 0.183 | 2.2 | 0.014 |
| KCNMB1 | 0.276 | 1.32 | 0.127 | 2.18 | 0.0145 |
| GSTA2 | -0.0933 | 0.911 | 0.044 | -2.12 | 0.017 |
| HSPB8 | 0.295 | 1.34 | 0.141 | 2.1 | 0.018 |
| TACR2 | 0.267 | 1.31 | 0.128 | 2.09 | 0.0185 |
| AKAP6 | 0.456 | 1.58 | 0.224 | 2.03 | 0.021 |
| HSPB7 | 0.214 | 1.24 | 0.106 | 2.02 | 0.022 |
| PNLIPRP2 | -0.0922 | 0.912 | 0.0464 | -1.98 | 0.0235 |
| ADAMDEC1 | -0.226 | 0.798 | 0.114 | -1.99 | 0.0235 |
| DES | 0.147 | 1.16 | 0.075 | 1.97 | 0.0245 |
| EPHA6 | 0.107 | 1.11 | 0.0548 | 1.96 | 0.025 |
| LIPJ | 0.118 | 1.13 | 0.0618 | 1.91 | 0.028 |
| C2CD4A | -0.205 | 0.815 | 0.11 | -1.86 | 0.0315 |
| UGT2A3 | -0.115 | 0.891 | 0.0622 | -1.85 | 0.032 |
| PDK4 | 0.371 | 1.45 | 0.207 | 1.79 | 0.037 |
| RNASE7 | -0.0773 | 0.926 | 0.0432 | -1.79 | 0.037 |
| MYLK | 0.306 | 1.36 | 0.172 | 1.78 | 0.0375 |
| HAND2 | 0.179 | 1.2 | 0.102 | 1.75 | 0.04 |
| PKHD1L1 | -0.117 | 0.889 | 0.0671 | -1.75 | 0.0405 |
| FLNC | 0.252 | 1.29 | 0.144 | 1.74 | 0.0405 |
| AFP | -0.0891 | 0.915 | 0.0513 | -1.73 | 0.0415 |
| PRPH | 0.187 | 1.21 | 0.109 | 1.71 | 0.0435 |
| CASQ2 | 0.111 | 1.12 | 0.065 | 1.71 | 0.044 |
| SCN11A | 0.228 | 1.26 | 0.133 | 1.71 | 0.044 |
| VIP | 0.154 | 1.17 | 0.0904 | 1.71 | 0.044 |
| PRELP | 0.197 | 1.22 | 0.116 | 1.7 | 0.044 |
| ACSM4 | -0.0783 | 0.925 | 0.0468 | -1.67 | 0.047 |

**Table S5 Multivariate Cox regression analysis for DEGs associated with OS**

| Symbol | coef | Pr(>\|z\|) | HR | Lower 95%CI | Upper 95%CI |
| --- | --- | --- | --- | --- | --- |
| ADAMDEC1 | 1.042 | 0.01991 | 2.8349 | 1.179055 | 6.816 |
| ASB2 | 2.52611 | 0.00146 | 12.5048 | 2.638705 | 59.2603 |
| FLNC | -1.41038 | 0.03879 | 0.244 | 0.064048 | 0.9299 |
| GPR15 | -0.37357 | 0.00256 | 0.6883 | 0.539949 | 0.8773 |
| HAND2 | -1.06614 | 0.02337 | 0.3443 | 0.136999 | 0.8655 |
| PRPH | 0.7166 | 0.04085 | 2.0475 | 1.030253 | 4.069 |
| RNASE7 | -0.37759 | 0.0186 | 0.6855 | 0.500555 | 0.9388 |
| TCL1A | -0.63524 | 0.00042 | 0.5298 | 0.372232 | 0.7541 |
